# The extent to which child and parent report RCADS, sMFQ, SDQ and child report KIDSCREEN identify the same young people as at risk of mental health conditions

**DOI:** 10.1192/bjp.2025.5

**Published:** 2025-03-26

**Authors:** Nazneen Nazeer, Jenny Parker, Lauren Cross, Sophie Epstein, Jessica Penhallow, Tamsin Newlove-Delgado, Johnny Downs, Tamsin Ford

**Affiliations:** Department of Psychiatry, University of Cambridge, Cambridge, UK; Department of Psychiatry, University of Cambridge, Cambridge, UK; North East London Foundation Trust, London, UK; MRC Epidemiology Unit, University of Cambridge, Cambridge, UK; Department of Psychiatry, University of Cambridge, Cambridge, UK; Institute of Psychiatry of Psychology, Psychiatry and Neuroscience, Department of Psychological Medicine, King’s College London, London, UK; South London and Maudsley NHS Foundation Trust, UK; Department of Child and Adolescent Psychiatry, King’s College London, London, UK; College of Medicine and Health, University of Exeter, Exeter, UK; Department of Child & Adolescent Psychiatry and Child and Adolescent lead, Centre for Translational Informatics, King’s College London, London, UK; Department of Psychiatry, University of Cambridge, Cambridge, UK

**Keywords:** correlation, SDQ, sMFQ, RCADS, KIDSCREEN

## Abstract

**Background:**

We rely heavily on cut-points of brief measures of psychological distress in research and clinical practice to identify those at risk of mental health conditions, however, few studies have compared the performance of different scales.

**Aim:**

To determine the extent to which child and parent report Strength and Difficulty Questionnaire (SDQ), Revised Children’s Anxiety and Depression Scale (RCADS), short-Moods and Feeling Questionnaire (sMFQ) and child report KIDSCREEN correlated and identified the same respondents above cut-points and at risk of mental health conditions.

**Methods:**

A cross-sectional survey was conducted among 231 children aged 11-16 years who completed all the above measures administered via a mobile App, MyJournE, and 289 parents who completed SDQ, RCADS and sMFQ.

**Results:**

The psychopathology measures identified similar proportions of young-people as above cut-point and at risk of depression (child-report = 14.7% RCADS, 19.9% sMFQ, parent-report = 8.7% RCADS, 12.1% sMFQ), anxiety (child-report = 24.7% RCADS, 26.0% SDQ emotional subscale, parent-report = 20.1% RCADS, 26% SDQ emotional subscale) and child-report internalising problems (26.8% RCADS, 29.9% SDQ). Despite strong correlations between measures (child-report = 0.77 to 0.84, and parent-report = 0.70 to 0.80, between SDQ, sMFQ and RCADS) and expected directions of correlation with KIDSCREEN and SDQ subscales, Kappas indicates moderate to substantial agreement between measures. Measures did not consistently identify the same children; half (n=36, 46%) of those on child-report and a third (n=30, 37%) on parent-report, scoring above cut-point for either SDQ emotional subscale, RCADS total or sMFQ, scored above cut-point on all of them. Only half (n=46, 54%) of the children scored above the cut-point on child-report by the SDQ-Internalising and RCADS total scales.

**Conclusion:**

This study highlights the risk of using a screen to ‘rule-out’ potential psychopathology. Screens should not be used diagnostically and are best used together with broad assessment.

## Introduction

Many people who experience mental health conditions experience their first difficulties in childhood or adolescence (1). Although these difficulties tend to fluctuate in severity over time, the resulting impairment hampers development and undermines recovery. The importance of sound and suitable tools for practitioners to identify and monitor changes in mental health to support prevention, intervention, research and commissioning cannot be over-emphasized. We now have a wide range of generic and specific measures designed to evaluate mental health and well-being across community, clinical and research settings. However, selecting the best measure that can be used both in clinical practice as well as in service evaluation can be challenging (2). These tools must be psychometrically robust and data needs to be as complete as possible to generate accurate information when applied at system level (3) in order to drive improvements in service quality and outcomes(4). In contrast, such tools used during clinical encounters should provide a valid and reliable measure of improvement or deterioration on an individual basis to guide effective and efficient interventions.

### Specific Measures

Please see Supplementary information for more detail on the psychometric function of all the measures discussed below. The National Institute of Mental Health, Wellcome Trust and International Alliance Mental Health Research Funders agreed to a minimum set focused outcome measures for anxiety and depression and unanimously endorsed the Revised Child Anxiety Depression Scale (RCADS) (5). The International Consortium for Health Outcomes Measurement (ICHOM) also recommends RCADS as a standard outcome measure to evaluate symptoms of anxiety and depression in children and young people (6). The RCADS measures anxiety and depression among children aged 6 to 18 years; the original 47-item questionnaire was adapted from the Spence Children’s Anxiety Scale (SCAC) (7) with versions for parents and children to complete (8). Recognizing the need for a brief measure, RCADS was reduced to a 25-item scale (9). Both versions successfully discriminate between clinical samples of young people with a diagnosis of an anxiety or depressive disorder and community samples have demonstrated acceptable levels of validity (9) and reliability (8). RCADS was further reduced to an 11-item scale incorporating items in the anxiety subscale that related to DSM-5 anxiety-disorder symptoms for administration at school and primary-care settings (10). Similarly, the Mood and Feeling Questionnaire (MFQ) is recommended by the National Institute for Health and Care Excellence (NICE) as an adjunct to clinical judgement to monitor the treatment of children and young people with depression (NICE, 2019). It was also used in several UK-based randomized control trials; IMPACT(11), The Thinking Styles Trial (12) and PROMISE trial (13). The MFQ was first developed as a 33-item questionnaire designed to measure core depressive symptomology in children and adolescents aged 8 to 18 years old and later validated for children aged 6 to 19 years with parent and self-report versions(14,15).

### Generic Measures

General measures of psychopathology remain important; the current focus of funders on anxiety and depression risks overlooking other mental health conditions and comorbid difficulties that might influence treatment outcomes. Indeed, recent commentaries have suggested a transdiagnostic general psychopathology factor, best understood as a reflection of the extent of impairment or dysfunction in a person’s life, and a bi-factor model, which includes an internalising psychopathology factor characterized by an increased propensity to respond to stress and negative mood with maladaptive repetitive thinking (16). The Strength and Difficulty Questionnaire (SDQ) and KIDSCREEN questionnaire are two such generic tools that have been used extensively in clinical practice and research. The SDQ was designed to assess common childhood mental health conditions in clinical samples aged 4 to 17 years, with versions for parents, teachers and young people (17). The scale consists of 25 items, divided into five subscales that measure emotional symptoms, conduct problems, hyperactivity/inattention, peer relationship problems and prosocial behaviour. The SDQ is validated for completion by children aged 11 and older; careful testing suggests that the concepts and language used are too complex for younger children, or young adolescents with a low reading age (18). In contrast, KIDSCREEN focuses on health-related quality of life and was developed with inputs from children and validated for children as young as 8 years old, unlike most other general wellbeing scales, and is a self-report measure, completed by children to assess their health and wellbeing.

To our knowledge, there have been no head-to-head comparisons of how children and young-people and parents score across these measures applied simultaneously, particularly in terms of overlap in children identified as at high risk of having a mental health condition. Knowledge of how these measures relate to each other could prove useful for comparisons across datasets given they are all commonly used. Hence, we aimed to determine the extent to which the SDQ, RCADS and sMFQ identified the same children as at risk, defined as scoring above accepted cut points, and to determine the extent of correlation between these measures. We hypothesized that the SDQ-emotional (SDQ-E) and RCADS-Total (RCADS-T), plus the RCADS-Depression (RACDS-D) and sMFQ would have the greatest overlap. Likewise, we anticipated that the SDQ-internalising (SDQ-I) and emotional subscales would be more strongly correlated with the RCADS-T, plus the RCADS-D and sMFQ scores than the other subscales. Finally, in the child report, we expected that the school environment and social acceptance subscales of KIDSCREEN would be negatively correlated with all subscales except the SDQ-prosocial behaviour subscale, which would show a positive correlation.

## Method

### Participants

A cross-sectional, follow-on survey was conducted in children aged 11-16 years and parents, sampled from the participants in the Mental Health of Children and Young People (MHCYP) Survey Wave 2 follow-up which aimed to assess the mental health of children and young-people in England (19). The Covid-19 pandemic prompted follow-up surveys (20) of this sample, and the first (summer 2020) sought consent for contact to participate in mood and stress monitoring via a mobile App, MyJournE (21). A total of each 889 child and parent participants, were approached between mid-October and November, 2021 during partial school closures and lockdown in England, with twice weekly reminders to those who had not enrolled.

### Survey procedure

MyJournE is a secure and free app and e-platform co-designed with young-people and researchers at King’s College London(21). Participants were enrolled in the study via email from which they were able to access and download the MyJournE app. Participant as well as parental e-consents were embedded within the mobile app and MyJournE e-platform, respectively. Participants were sign-posted to carefully selected health resources throughout the project, which included both participant material (Ex: Participant Information Sheet) and ‘Support Pages’ in the app itself. The survey took approximately 15 minutes to complete.

The authors assert that all procedures contributing to this work comply with the ethical standards of the relevant national and institutional committees on human experimentation and with the Helsinki Declaration of 1975, as revised in 2013. All procedures involving human subjects/patients were approved by University of Cambridge Psychology Research Ethics Committee (PRE.2021.047).

### Measures

**RCADS** : MyJournE used the 11-item version of the RCADS (10). For children we selected a cut-point of ≥7.5 for anxiety, ≥8.5 for depression and ≥12.5 for total score and for parents ≥5.5 for anxiety, ≥6.5 for depression and ≥10.5 for total score based on the recent UK validation study (10).

**SDQ** : the self-report version was integrated into the app (22). We used the 4-band categorization of the SDQ scores (23). For total scores and sub-domains, (except for prosocial behaviour where “low” is the cut-point) “high” category (reflecting top 10% of abnormal scores) was set as the cut-point which correspond to a score of ≥ 18 for total difficulty (≥17 parent-report), ≥ 6 for emotional problems (≥5 parent-report), ≥5 for conduct problems (≥4 parent-report), ≥7 for hyperactivity (≥8 parent-report), ≥ 4peer problem (≥4 parent-report) and ≤ 5 for prosocial behaviour (≤6 parent-report). Child-report SDQ cut-points for internalising (emotional + peer problem) and externalising (hyperactivity + conduct problems) scales, which are not routinely used in clinical practice, were computed using the British Child and Adolescent Mental Health Survey 1999 dataset (24) and were determined at 90th percentile which corresponded to scores of ≥8 and ≥10 for internalising and externalising scales respectively.

**sMFQ** : the app incorporated the 13-item self-report version (25). We used the cut-point set at a score of ≥12 based on a validation study in New Zealand conducted among a similar aged sample of adolescents (26). For sMFQ parent-report we used a cut-point of ≥11 based on a validation study in UK community sample(27).

**KIDSCREEN**: the app included two domains of the child-report 52-item version; School environment, which reflected on the child’s wellbeing at school (6-items) and social acceptance, whether they were fearful of school and whether they were being bullied (3-items). As there were no recommended cut-points for each of these subscales, we used the 25th percentile, below which the health related quality of life (HRQoL) was considered poor as applied in a study that was conducted in six European countries including the UK (28). The score that corresponded to the 25th percentile for school environment was 60 from a range of 23-100 and 87 for social acceptance from a range of 20-100.

### Statistical analysis

Statistical analysis was carried out using IBM SPSS 22.0 software. We calculated the raw scores for each measure, explored their distribution (mean and standard deviation (SD)); and calculated the proportions scoring above cut-points with 95% confidence intervals to allow comparison of estimates.

Differences between the App sample and the National sample for SDQ and the App sample and European norms for KIDSCREEN measures were analysed using one sample t-tests using the MHCYP follow-up 2021 (20) as the reference mean for the SDQ and the mean percentage scores from the European Norm data (29) for the KIDSCREEN. Suitable UK norms for RCADS and sMFQ were not available for comparison.

We constructed Venn diagrams to illustrate the overlaps among those identified as being at risk of mental health conditions because they scored above our selected cut-points between conceptually similar scales and subscales of SDQ, RCADS and sMFQ measures, and assessed chance-corrected agreement on at-risk caseness using the Kappa statistic. Finally, we assessed Pearson’s correlations between all four measures.

## Results

Of each 899 children and parents approached, 231 children and 289 parents participated in our survey giving a response rate of 26% and 32% respectively. The age of respondents ranged from 11-16 years (mean=13.92; SD=1.74), and 53.3% were girls. Compared to the National Sample the MyJournE sample reported statistically significantly higher mean scores for total difficulty (p <0.001), emotional problems (p<0.001), conduct problems and hyperactivity (p=0.002) and significantly lower mean prosocial behaviour scores (p=0.002) indicating that we recruited a subsample of children with poorer mental health. Sub-analysis of the genders revealed a statistically significant elevation of emotional problems only among boys(p<0.001), while girls showed significant elevation of scores in all domains except peer problems and significant lower mean prosocial behaviour scores (p<0.001) (Supplementary Table 1). In contrast, mean scores of school environment-related HRQoL in the App sample were significantly higher (p<0.001) compared to European norms(29) indicating a better HRQoL (Supplementary Table 2).

Table 1 describes the distribution of child and parent scores from the SDQ, RACDS, sMFQ and child-report KIDSCREEN and the proportion scoring above cut-point. Notably the number of children scoring above cut-point for SDQ total difficulty score (SDQ-T) (19.1% child-report and 16.6% parent-report) was lower than the RCADS-T (26.8 % child-report and 19% parent-report). According to the SDQ subscales, emotional problems were the most commonly reported difficulty by both children and parents. The number of children scoring above cut-point on SDQ-E was similar to the number scoring above cut-point on RCADS-T for child-report (26% and 26.8% respectively), although higher for parent-report (26% and 19% respectively). Almost twice as many children scored themselves as being at risk of internalising disorders (29.9%) compared to externalising disorders (15.2%). The number of children scoring above cut-point on sMFQ was higher than on the RCADS-D for both child-report (19.9% and 14.7% respectively) and parent-report (12.1% and 8.7% respectively).

Figure 1(i) provides a range of Venn Diagrams to illustrate how these measures compared with each other in the same children scoring above cut-point. Figure1a illustrated a moderate overlap (n=46; 54.1%) of all children scoring above cut-point by the RCADS-T and SDQ-I scale. Figure 1(i)(b) illustrates how, despite similar frequency of children scoring above cut-point, the RCADS-T (n=62) and SDQ-E (n=60) only identified 47 children in common with each other, with RCADS-T ‘missing’ 13 children identified by SDQ-E and similarly SDQ-E ‘missing’ 15 children identified by RCADS-T. Figure 1(i)(c) highlights that out of a total of 63 children who scored above cut-point on RCADS-D or RCADS -Anxiety (RCADS-A) or the sMFQ, 28 (44%) children were identified by both RCADS-D and RCADS-A reflecting the common comorbidity between these two conditions. The overlap of children scoring above cut-point between SDQ-E and RCADS-A was greater than depression on RCADS-D, which is not surprising as SDQ-E comprises four questions about fears and worries and only one about sadness. In Figure 1(i)(d), using the general scale, SDQ-E ‘missed’ 15 children (20%) who scored above cut-point on the RCADS-A and RCADS-D. Conversely, the specific RCADS scales do not identify 12 children (16%) who scored above cut-point on the SDQ. Similarly, sMFQ did not identify above cut-point 22 children (32%) that the RCADS-D did, whereas in the reverse comparison, the RCADS-D ‘missed’ 5 children scoring above cut-point (7%) which the sMFQ did (Figure 1(i)c).

Figure 1(ii) provides a similar representation using Venn Diagrams for parent-report. Figure 1(ii)(a) highlights how the SDQ-E identifies nearly all children identified by sMFQ, and the large proportion of those identified by the RCADS-T (91%). In Figure 1(ii) b, using the general scale, SDQ-E ‘missed’ 11 children (12%) who scored above cut-point on the RCADS-A and RACDS-D. Conversely, both RCADS subscales do not identify 24 children (28%) who scored above cut-point on the SDQ-E. Figure 1(ii)(c) shows the overlap in children scoring above cut-point between sMFQ and RCADS-D is modest. Considering total of 43 cases identified by both, the sMFQ ‘misses’ 8 children (19%) scoring above cut-point on RCADS-D, while RCADS-D ‘misses’ 18 children (41%) which the sMFQ did.

Table 2 depicts correlations of RCADS and sMFQ scores with SDQ scores for both raters. Despite the modest overlap in children scoring above cut-point between measures, we observed moderate to high correlations across all measures. Predictably, SDQ-E were significantly, strongly and positively correlated with measures of anxiety (RCADS-A) and depression (RCADS-D & sMFQ) and RCADS-T scores. Similarly, the child-report SDQ-I scores, which is a combination of both emotional and peer problems, demonstrated significant, positive and equally strong correlations with anxiety and depression measures, and these were not markedly different to the emotional subscale alone. Notably, the SDQ-T, which also incorporates attention and behavioural difficulties, demonstrated correlations that were only very slightly weaker than the emotional and internalising subscales as indicated by the overlapping confidence intervals. Similarly, there were moderate and positive correlations of conduct and hyperactivity domains with RCADS and sMFQ scales, implying either considerable comorbidity or conceptual overlap. As expected, prosocial behaviour scores showed significant but negative correlations with anxiety and depression scores.

Considering correlations between psychopathology scales and KIDSCREEN scale, we found moderate to strong correlations between total and subscales scores of all measures (SDQ, RCADS and sMFQ) with the school environment; positive for prosocial behaviour and negative for all the others. Although, social acceptance subscale showed a similar pattern, correlations with prosocial behaviour, and the correlations with conduct and hyperactivity subscales were surprisingly small and not statistically significant (Table 3).

Kappa statistics was performed to determine the chance-corrected agreement between scales/subscales measuring similar psychological constructs and ranged between 0.52-0.77 (p<0.001), showing moderate to substantial strength of agreement (Table 4).

## Discussion

We aimed to explore the extent to which four common measures of child psychopathologies related to each other, particularly in identifying the same children above recommended cut-points. We found moderate to high correlations between measures as expected, but surprisingly, relatively modest overlap in the children identified as at risk of mental health conditions even between conceptually similar measures. Our findings also revealed surprisingly low correlations between hyperactivity, conduct problems and prosocial behaviour with social acceptance.

We anticipated that the SDQ would identify a greater number of children at risk of mental health difficulty than RCADS or sMFQ, given that it assesses a broader range of problems. However, our study revealed contrasting findings. Goodman et al, 2010 in their study cautioned against the use of the five-factor structure of the SDQ in low-risk, community samples owing to its poor discriminant validity between emotional and peer problem domains. Instead, a more conservative approach of using internalising and externalising subscales was recommended (30). Our findings fit this recommendation as the SDQ-T cut-point identified a lower number of children than the RCADS-T whereas, the SDQ emotional or internalising subscale identified similar numbers.

Our study reported very strong, positive correlations between the SDQ emotional, internalising and total scores with RCADS and sMFQ scores. These findings suggest the usefulness of a general questionnaire such as the SDQ in indicating the presence of potential anxiety or depressive disorder, as well as the advantage of a broader range of questions that could denote the presence of disorders in other domains (31).

Although both SDQ-I and RCADS-T identified similar proportions of young people as possible cases, we observed only modest overlap *between* the two internalising scales despite their conceptual similarity. Differences possibly emerge from the structured components; while both scales are similarly structured to measure anxiety, RCADS consists of an exclusive subcomponent to measure depression while the SDQ-I contains one question about sadness and combines the emotional and peer relationship problem subscales. Furthermore, the peer problem items, which include questions that assess preference for solitude, the lack of friends, being unpopular with other children, better relationships with adults than children and being bullied, do not exclusively map onto internalising difficulties.

Our child-report sample reported higher levels of internalising problems than externalising problems, which is consistent with the National Survey Programme (Mental Health of Children and Adolescents of Great Britain and MHCYP surveys) that indicated a significant increase in emotional disorders among 5–15-year-olds between 1999, 2004 and 2017 while behavioural and hyperactivity disorders remained broadly stable (19). The proportion of young-people reporting difficulties in focusing attention, however, is markedly higher than these earlier national surveys of psychiatric disorders would predict. These findings add to contradictory reports about the impact of Covid-19 pandemic and its resultant restrictions on these same scales (32).

In our sample, sMFQ identified more children above cut-point compared to the RCADS-D. Evidence shows that the two additional symptom impact items related to distress and interference at school contribute to improved accuracy of the 11-item RCADS (10) and is consistent with a plethora of previous research that highlights the superiority of including impact items over symptom items alone when estimating the prevalence of clinically impairing mental health problems in young-people (33–35). In addition, Radez and colleagues (2021) demonstrated that for 11-item RCADS, adolescent report combined with parent report generated the most accurate results (10), as have other similar studies of multiple versus single informants(36–38). The RCADS is highly sensitive to gender differences (39) while sMFQ has been shown to measure depression equivalently in males and females (40). We chose to use a common cut-off disregarding gender in our study for the RCADS, which may have contributed to its underperformance, but it remains interesting to consider why the RCADS requires different thresholds for girls and boys when few other psychopathology measures do. As expected, there was a greater overlap between RCADS-D and sMFQ than between RCADS-D and RCADS-A. This was also reported by Radez et al, 2021 who established a high convergent validity between these depression scales with moderate to high correlation scores (10). These authors also detected moderate correlations between sMFQ scores and RCADS-A reflective of the known common comorbidity between anxiety and depressive symptoms (19), which was also evident in our study.

Our study replicates results from prior work establishing the inverse associations between adverse school environment with anxiety, depression, internalising and externalising problems in children (41,42); a Finnish, population-based longitudinal study revealed the close association of a poor classroom climate with internalising behaviour (43). Similarly, social acceptance displayed inverse associations with the SDQ-I, RCADS-A, RCADS-D and sMFQ which are consistent with previous studies (44,45). However, contrary to our hypothesis social acceptance neither demonstrated a significant negative correlation with behavioural problems, nor a significant positive correlation with prosocial behaviour. The 3-items of the social acceptance domain of the KIDSCREEN questionnaire focuses on identifying children who are victims of bullying. Evidence suggests that victims of bullying (implying low social acceptance) are at greater risk of suffering from internalising symptoms while behavioural problems are more relevant for bullies(46–48). A good classroom climate with a positive student-teacher relationship may have had a cushioning effect against bullying(49) which may have not affected prosocial behaviour adversely. Alternatively, the unusual social and school circumstances during data collection, when schools were partially open but with social isolation for those groups exposed to infection, may have impacted on both the school experience and reporting.

Our findings emphasize that no measure is perfect for all situations. This issue has led to a plethora of scales to measure the same construct. One possible solution is the use of computer-adapted-testing which pools a large collection of items from various instruments calibrated using item response theory to generate short personalised assessments (50). Instead of studying whole instruments separately, these models enable identifying useful items and evaluating the extent of overlap between instruments and the best set of items for specific assessment purposes.

### Strengths and limitations

Our study benefitted from a moderately-large sample of young-people who completed these commonly used mental-health scales simultaneously. The sample who took part was a self-selecting group from within another study and differed from the wider study group indicating poorer mental health. This selection bias towards those with higher psychopathology and a tendency to score above cut-points in screening questionnaires may relate to the primary purpose of the study, which invited people to monitor their mood and mental health over a period. However, in relation to our research question, an in-group comparison, and generalizability to the general population is not essential. Indeed, the group more closely represent those who may seek clinical help, which increase the relevance of our findings to the clinical population and considered a strength of the study. Inevitably there are some methodological limitations. Firstly, the MFQ and RCADS applied were both short versions and evidence of their psychometric properties and measures of diagnostic accuracy are scarcer than the longer, original versions. However, the shorter version of the RCADS has been recommended when brevity is required as is often the case in clinical practice or large surveys (5). Secondly, the lack of impact scores on the SDQ and RCADS restricted taking into account the overall distress and level of functional impairment when reaching a decision on cut-points. The choice of cut-points for each scale will have influenced the overlap, but we used those most strongly endorsed for each measure.

## Conclusions

These questionnaires, particularly SDQ and RCADS, are routinely used in clinical CAMHS settings in the UK, at triage and initial assessment. Despite strong correlations between these commonly used measures, there is surprisingly modest overlap between children identified above cut-point by child or parent report. Taken at individual child level a clinician cannot be wholly confident that if a child scores below cut-off on a screen, by child or parent report, that they will score below cut-off in other screens correlating to similar psychopathologies. This highlights the risk of using a screen to ‘rule-out’ potential psychopathology at triage without further assessment. Screens should not be used diagnostically, but are best used in conjunction with broad assessment and contain valuable information regardless of whether cut-point is reached. Researchers, clinicians and policymakers must consider the relative strengths and weaknesses of particular measures in relation to their intended purpose.

## Supplementary Material

Supplementary Materials

## Figures and Tables

**Figure 1(i):**
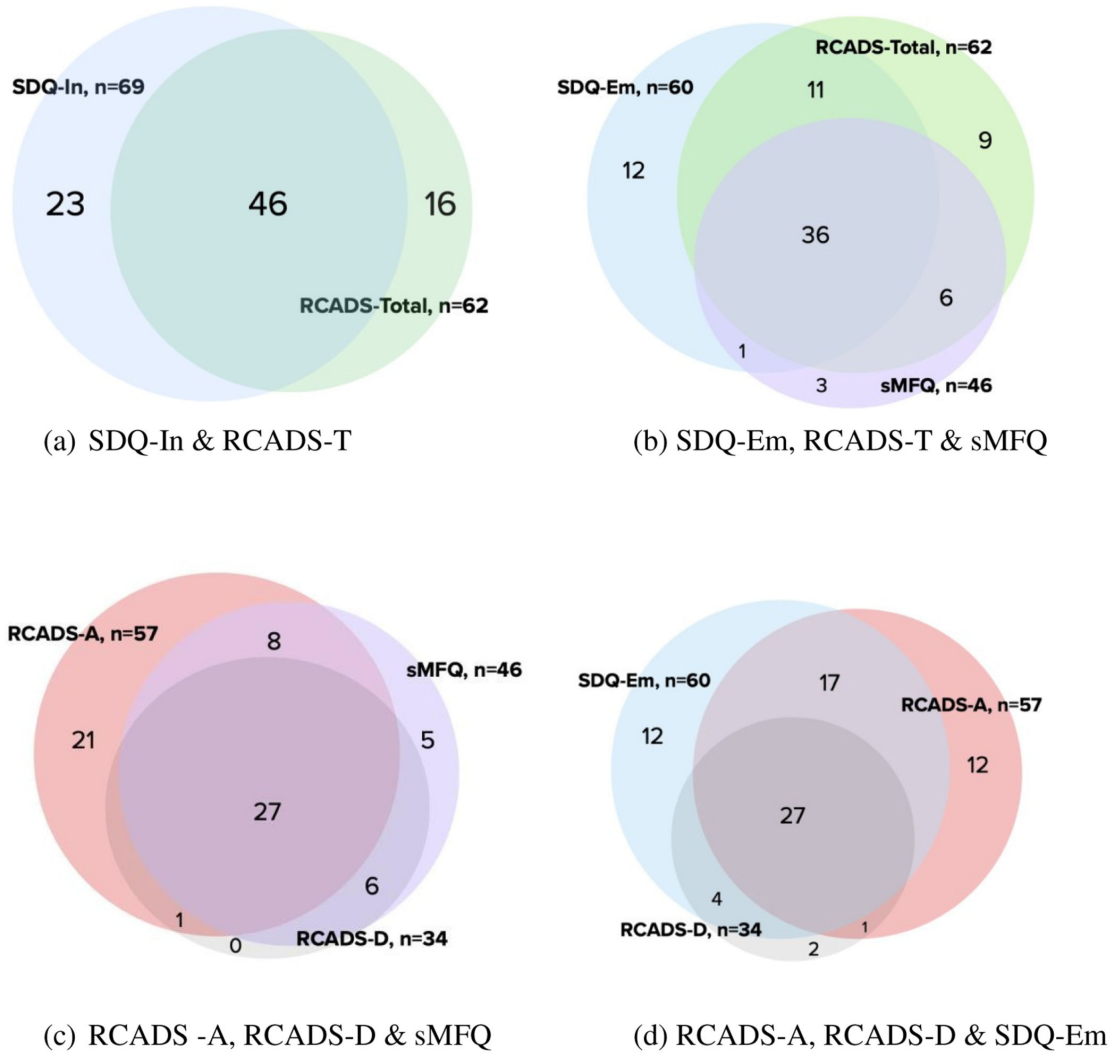
Panel comparing the children self-report above cut points on (a) SDQ-In & RCADS-T; (b) SDQ-Em, RCADS-T & sMFQ; (c) RCADS-D, RCADS-A & sMFQ; (d) SDQ-Em, RCADS-A & RCADS-D (Representative, not to scale) N=231, SDQ-Em= SDQ Emotional, SDQ-In= Internalizing, RCADS-T= RCADS-Total, RCADS-A= RCADS Anxiety, RCADS-D=RCADS Depression

**Figure 1(ii):**
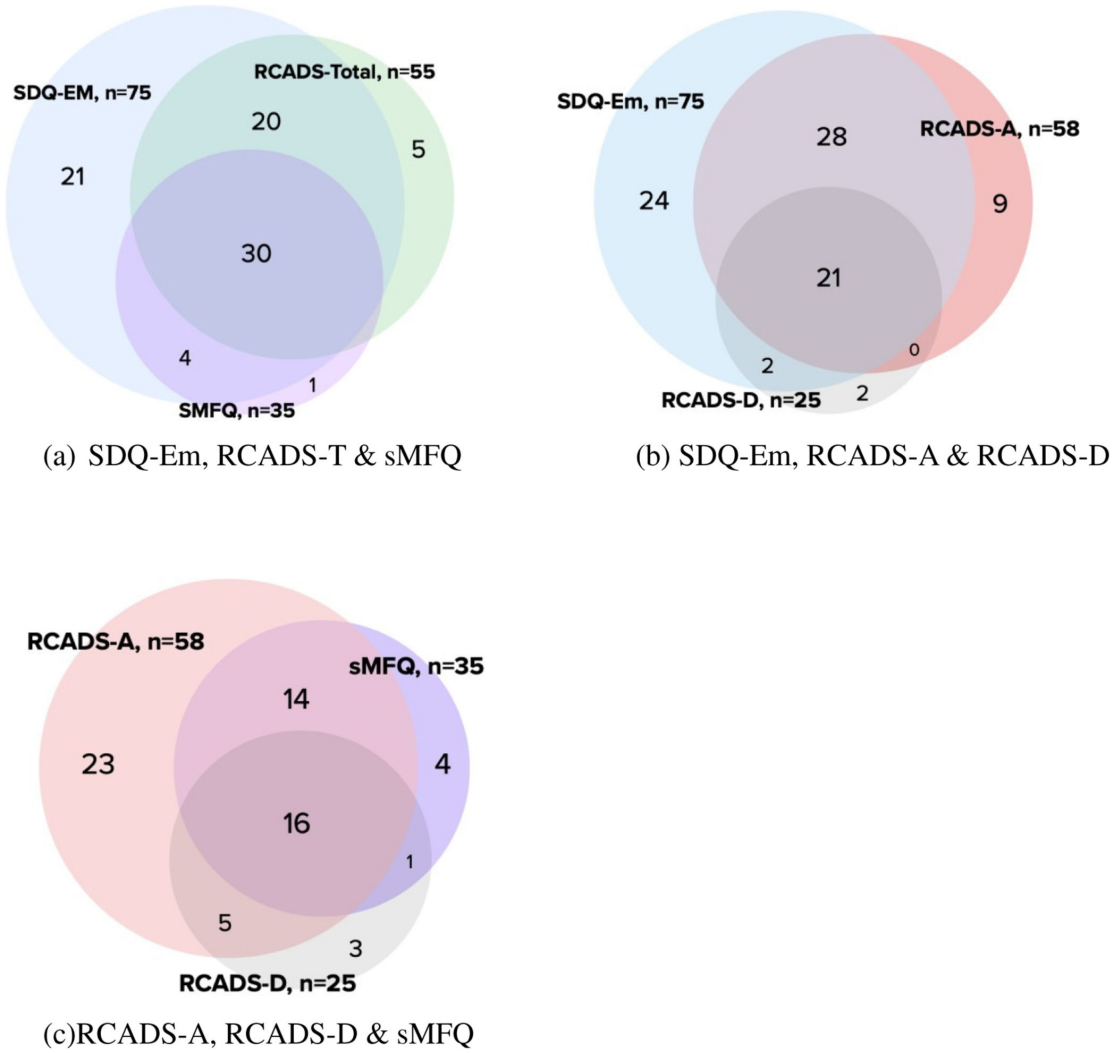
Panel comparing parent report above cut points on (a) SDQ-Em, RCADS-T &sMFQ; (b) SDQ-Em, RCADS-A & RCADS-D; (c) RCADS-D, RCADS-A & sMFQ (Representative, not to scale) N=289, SDQ-Em= SDQ Emotional, RCADS-T= RCADS-Total, RCADS-A= RCADS Anxiety, RCADS-D=RCADS Depression

**Table 1:** Proportion of children scoring above cut point, at risk of mental health conditions on the SDQ, RCADS, sMFQ and KIDSCREEN and distribution of scores, by child and parent report.

	Child Report, at risk of mental health conditions ^a^			Parent Report, at risk of mental health conditions ^b^		
	Number (%)	Mean	SD	Number (%)	Mean	SD
**SDQ**						
Total difficulty	44 (19.1)	11.4	6.8	48 (16.6)	9.4	6.7
Emotional problems	60 (26.0)	3.6	2.7	75 (26.0)	3.0	2.7
Conduct problems	18 (7.8)	1.6	1.8	27 (9.3)	1.3	1.5
Hyperactivity	50 (21.7)	4.1	2.7	21 (7.3)	3.3	2.6
Peer problems	40 (17.3)	1.9	1.8	59 (20.4)	1.9	2.0
Pro-social behaviour	27 (11.7)	7.6	1.8	70 (24.2)	7.8	2.0
Internalising scale	69 (29.9)	5.5	4.0	-	-	-
Externalising scale	35 (15.2)	5.8	3.9	-	-	-
**RCADS**						
Anxiety	57 (24.7)	5.3	4.3	58 (20.1)	3.7	3.4
Depression	34 (14.7)	4.5	3.6	25 (8.7)	2.9	2.8
Total (Anxiety + Depression)	62 (26.8)	9.8	7.5	55 (19.0)	6.6	5.8
**sMFQ**						
Depression	46 (19.9)	6.5	0.4	35 (12.1)	4.0	5.1
**KIDSCREEN**						
School environment	52 (22.5)	71.2	16.6	-	-	-
Social acceptance	47 (20.3)	90.4	13.9	-	-	-

*Child report N=231, Parent report N=289, SD=Standard Deviation*

a Cut point child report for SDQ (scoring at and above “high” corresponding to scores ≥ 18 for total difficulty, ≥ 6 for emotional problems, ≥ 5 for conduct problems, ≥ 7 for hyperactivity, ≥4 peer problem, ≤ 5 for prosocial behaviour [prosocial behaviour scores inversely and not included in total score], ≥8 for internalising and ≥ 10 for externalising subscales); for RCADS (disregarding gender, ≥7.5 for anxiety, ≥8.5 for depression and ≥12.5 for Total score); for sMFQ (≥ 12) and for KIDSCREEN (scores <25th percentile corresponding to a score of 60.0 for school environment and 86.6 for social acceptance)

b Cut point parent report for SDQ (scoring at and above “high” corresponding to scores ≥ 17 for total difficulty, ≥ 5 for emotional problems, ≥ 4 for conduct problems, ≥ 8 for hyperactivity, ≥4 peer problem, ≤ 6 for prosocial behaviour [prosocial behaviour scores inversely and not included in total score], for RCADS (disregarding gender, ≥5.5 for anxiety, ≥6.5 for depression and ≥10.5 for Total score); for sMFQ (≥ 11)

**Table 2:** Pearson’s Correlation of anxiety, depression and total scores (from RCADS) and depression scores (from sMFQ) with total and subdomains of SDQ scores

Child report SDQ subdomains	RCADS, Child report						sMFQ, Child report	
	Anxiety score		Depression score		Total scores		Depression scores	
	^a^CC (r)	95%CI	CC (r)	95%CI	CC (r)	95%CI	CC (r)	95%CI
**Total difficulty scores**	0.70*	0.64-0.77	0.73*	0.66-0.79	0.76*	0.71-0.81	0.73*	0.68-0.79
**Emotional problem scores**	0.80*	0.75-0.85	0.77*	0.71-0.82	0.84*	0.79-0.87	0.77*	0.71-0.82
**Conduct problem scores**	0.31*	0.19-0.42	0.36*	0.07-0.48	0.35*	0.23-0.47	0.38*	0.24-0.51
**Hyperactivity problem scores**	0.41*	0.29-0.52	0.44*	0.33-0.54	0.45*	0.35-0.55	0.48*	0.37-0.57
**Peer problems**	0.52*	0.40-0.64	0.56*	0.45-0.65	0.58*	0.45-0.68	0.53*	0.43-0.63
**Prosocial behaviour scores**	-0.15**	-0.29- -0.01	-0.24*	-0.36- -0.10	-0.2**	-0.34- -0.07	-0.21**	-0.33- -0.82
**Internalising problems**	0.78*	0.72-0.84	0.78*	0.72-0.83	0.83*	0.78-0.87	0.76*	0.69-0.82
**Parent report SDQ subdomains**	**RCADS, Parent report**						**sMFQ, Parent report**	
	**Anxiety score**		**Depression score**		**Total scores**		**Depression scores**	
	^a^CC (r)	95%CI	CC (r)	95%CI	CC (r)	95%CI	CC (r)	95%CI
**Total difficulty scores**	0.69*	0.63-0.75	0.67*	0.61-0.73	0.74*	068-0.78	0.77*	0.71-0.81
**Emotional problem scores**	0.77*	0.73-0.82	0.70*	0.64-0.76	0.80*	0.75-0.84	0.72*	0.66-0.77
**Conduct problem scores**	0.31*	0.20-0.41	0.41*	0.31-0.51	0.38*	0.28-0.48	0.54*	0.46-0.62
**Hyperactivity problem scores**	0.38*	0.28-0.46	0.34*	0.23-0.44	0.39*	0.29-0.48	0.47*	0.38-0.56
**Peer problems**	0.56*	0.48-0.64	0.56*	0.48-0.64	0.61*	0.53-0.68	0.57*	0.49-0.65
**Prosocial behaviour scores**	-0.34*	-0.44- -0.23	-0.40*	-0.49- -0.30	-0.4*	-0.49- -0.29	-0.45*	-0.54- -0.36
**sMFQ**	0.73*	0.67-0.78	0.77*	0.72-0.82	0.83*	0.76-0.84	0.76*	0.69-0.82

** Significant at p<0.05 level

* p<0.001 level

a CC=Correlation coefficient

**Table 3:** Correlation between school environment & social acceptance (KIDSCREEN scores) with SDQ, RCADS and sMFQ, child report

Parameter	School environment		Social acceptance	
	? CC (r)	95%CI	CC (r)	95%CI
**Total difficulty (SDQ)**	-0.65*	-0.73- -0.56	-0.4*	-0.5- -0.2
**Emotional problems (SDQ)**	-0.55*	-0.64- -0.44	-0.4*	-0.5- -0.3
**Conduct problems (SDQ)**	-0.47*	-0.57- -0.35	-0.1	-0.2- -0.05
**Hyperactivity (SDQ)**	-0.51*	-0.61- -0.41	-0.1	-0.2- -0.02
**Peer problems (SDQ)**	-0.41*	-0.51- -0.28	-0.5*	-0.6- -0.4
**Pro-social behaviour (SDQ)**	0.33*	0.19-0.45	0.01	-0.10-0.14
**Internalising problems (SDQ)**	-0.55*	-.65- -0.45	-0.5*	-0.6- -0.4
**Anxiety (RCADS)**	-0.50*	-6.0- -0.38	-0.5*	-0.6- -0.4
**Depression (RCADS)**	-0.61*	-0.68- -0.52	-0.4*	-0.5- -0.3
**Total (RCADS)**	-0.58*	-0.67- -0.48	-0.5*	-0.6- -0.4
**Depression (sMFQ)**	-0.61*	-0.68-0.51	-0.4*	-0.5- -0.3

* Significant at p<0.001 level

a CC=Correlation coefficient

**Table 4:** Chance corrected agreement between scales

Scales	Kappa	95% Confidence Interval	Strength of agreement
**Child Report Scales**			
**RCADS-A and SDQ-Em**	0.66	0.54-0.78	Substantial
**RCADS-T and SDQ-In**	0.59	0.47–0.71	Moderate
**RCADS-T and SDQ-Em**	0.69	0.50–0.81	Substantial
**RCADS-T and sMFQ**	0.71	0.61-0.81	Substantial
**RCADS-D and sMFQ**	0.77	0.65–0.89	Substantial
**SDQ-Em and sMFQ**	0.61	0.49-0.73	Substantial
**Parent Report Scales**			
**RCADS-A and SDQ-Em**	0.66	0.56–0.76	Substantial
**RCADS-T and SDQ-Em**	0.70	0.60–0.80	Substantial
**RCADS-T and sMFQ**	0.61	0.49–0.73	Substantial
**RCADS-D and sMFQ**	0.52	0.36–0.68	Moderate
**SDQ-Em and sMFQ**	0.54	0.42–0.66	Moderate

SDQ-Em= SDQ Emotional, SDQ-In= Internalizing, RCADS-T= RCADS-Total, RCADS-A= RCADS Anxiety, RCADS-D=RCADS Depression, sMFQ=Short Mood and Feeling Questionnaire

## Data Availability

Available on request from Prof. Tamsin Ford- tjf52@medschl.cam.ac.uk (Senior and corresponding author).

## Abbreviations

SDQ: Strength and Difficulty Questionnaire
sMFQ: short Mood and Feeling Questionnaire
RCADS: Revised Childrens’ Anxiety and Depression Scale
