# The extent to which child- and parent-report Revised Children’s Anxiety and Depression Scale, short Mood and Feeling Questionnaire, Strength and Difficulty Questionnaire and child-report KIDSCREEN identify the same young people as at risk of mental health conditions

**DOI:** 10.1192/bjp.2025.5

**Published:** 2026-03

**Authors:** Nazneen Nazeer, Jenny Parker, Lauren Cross, Sophie Epstein, Jessica Penhallow, Tamsin Newlove-Delgado, Johnny Downs, Tamsin Ford

**Affiliations:** Department of Psychiatry, University of Cambridge, Cambridge, UK; North East London Foundation Trust, London, UK; Child and Adolescent Mental Health Services, South London and Maudsley NHS Foundation Trust, London, UK; Department of Child & Adolescent Psychiatry and Child and Adolescent Lead, Centre for Translational Informatics, King’s College London, London, UK; College of Medicine and Health, University of Exeter, Exeter, UK; Cambridgeshire and Peterborough NHS Foundation Trust, Cambridge, UK

**Keywords:** Correlation, Strength and Difficulty Questionnaire, short Mood and Feeling Questionnaire, Revised Children’s Anxiety and Depression Scale, KIDSCREEN

## Abstract

**Background:**

We rely heavily on cut-off points of brief measures of psychological distress in research and clinical practice to identify those at risk of mental health conditions; however, few studies have compared the performance of different scales.

**Aim:**

To determine the extent to which the child- and parent-report Strength and Difficulty Questionnaire (SDQ), Revised Children’s Anxiety and Depression Scale (RCADS), short Mood and Feeling Questionnaire (sMFQ) and child-report KIDSCREEN correlated and identified the same respondents above cut-off points and at risk of mental health conditions.

**Method:**

A cross-sectional survey was conducted among 231 children aged 11–16 years and 289 parents who completed all the above measures administered via a mobile app, MyJournE, including the SDQ, RCADS and sMFQ.

**Results:**

The psychopathology measures identified similar proportions of young people as above the cut-off point and at risk of depression (child report 14.7% RCADS, 19.9% sMFQ, parent report 8.7% RCADS, 12.1% sMFQ), anxiety (child report 24.7% RCADS, 26.0% SDQ-Emotional subscale, parent report 20.1% RCADS, 26% SDQ-Emotional subscale) and child-report internalising problems (26.8% RCADS, 29.9% SDQ). Despite strong correlations between measures (child report 0.77–0.84 and parent report 0.70–0.80 between the SDQ, sMFQ and RCADS) and expected directions of correlation with KIDSCREEN and SDQ subscales, kappa values indicate moderate to substantial agreement between measures. Measures did not consistently identify the same children; half (*n* = 36, 46%) of those on child report and a third (*n* = 30, 37%) on parent report, scoring above the cut-off point for the SDQ-Emotional subscale, RCADS total or sMFQ, scored above the cut-off point on all of them. Only half (*n* = 46, 54%) of the children scored above the cut-off point on child report by the SDQ-Internalising and RCADS total scales.

**Conclusion:**

This study highlights the risk of using a screening test to ‘rule out’ potential psychopathology. Screening tests should not be used diagnostically and are best used together with broad assessment.

Many people who experience mental health conditions experience their first difficulties in childhood or adolescence.^1^ Although these difficulties tend to fluctuate in severity over time, the resulting impairment hampers development and undermines recovery. The importance of sound and suitable tools for practitioners to identify and monitor changes in mental health to support prevention, intervention, research and commissioning cannot be overemphasised. We now have a wide range of generic and specific measures designed to evaluate mental health and well-being across community, clinical and research settings. However, selecting the best measure that can be used both in clinical practice and in service evaluation can be challenging.^2^ These tools must be psychometrically robust and data need to be as complete as possible to generate accurate information when applied at the system level^3^ to drive improvements in service quality and outcomes.^4^ In contrast, such tools used during clinical encounters should provide a valid and reliable measure of improvement or deterioration on an individual basis to guide effective and efficient interventions.

## Specific measures

Please see supplementary information (available at https://doi.org/10.1192/bjp.2025.5) for more detail on the psychometric function of all the measures discussed below. The National Institute of Mental Health, Wellcome Trust and International Alliance Mental Health Research Funders agreed to a minimum set of focused outcome measures for anxiety and depression and unanimously endorsed the Revised Children’s Anxiety and Depression Scale (RCADS).^5^ The International Consortium for Health Outcomes Measurement (ICHOM) also recommends the RCADS as a standard outcome measure to evaluate symptoms of anxiety and depression in children and young people.^6^ The RCADS measures anxiety and depression among children aged 6–18 years; the original 47-item questionnaire was adapted from the Spence Children’s Anxiety Scale (SCAC),^7^ with versions for parents and children to complete.^8^ Recognising the need for a brief measure, the RCADS was reduced to a 25-item scale.^9^ Both versions successfully discriminate between clinical samples of young people with a diagnosis of an anxiety or depressive disorder, and community samples have demonstrated acceptable levels of validity^9^ and reliability.^8^ The RCADS was further reduced to an 11-item scale incorporating items in the anxiety subscale that related to DSM-5 anxiety-disorder symptoms for administration at school and in primary-care settings.^10^ Similarly, the Mood and Feeling Questionnaire (MFQ) is recommended by the National Institute for Health and Care Excellence (NICE) as an adjunct to clinical judgement to monitor the treatment of children and young people with depression (NICE, 2019). It was also used in several UK-based randomised control trials: IMPACT,^11^ The Thinking Styles Trial^12^ and the PROMISE trial.^13^ The MFQ was first developed as a 33-item questionnaire designed to measure core depressive symptomology in children and adolescents aged 8–18 years old and later validated for children aged 6–19 years with parent and self-report versions.^14,15^

## Generic measures

General measures of psychopathology remain important; the current focus of funders on anxiety and depression risks overlooking other mental health conditions and comorbid difficulties that might influence treatment outcomes. Indeed, recent commentaries have suggested a transdiagnostic general psychopathology factor, best understood as a reflection of the extent of impairment or dysfunction in a person’s life, and a bi-factor model, which includes an internalising psychopathology factor characterised by an increased propensity to respond to stress and negative mood with maladaptive repetitive thinking.^16^ The Strength and Difficulty Questionnaire (SDQ) and KIDSCREEN questionnaire are two such generic tools that have been used extensively in clinical practice and research. The SDQ was designed to assess common childhood mental health conditions in clinical samples aged 4–17 years, with versions for parents, teachers and young people.^17^ The scale consists of 25 items, divided into five subscales that measure emotional symptoms, conduct problems, hyperactivity/inattention, peer relationship problems and prosocial behaviour. The SDQ is validated for completion by children aged 11 and older; careful testing suggests that the concepts and language used are too complex for younger children, or young adolescents with a low reading age.^18^ In contrast, KIDSCREEN focuses on health-related quality of life (HRQoL) and was developed with inputs from children and validated for children as young as 8 years old, unlike most other general well-being scales, and is a self-report measure, completed by children to assess their health and well-being.

To our knowledge, there have been no head-to-head comparisons of how children and young people and parents score across these measures applied simultaneously, particularly in terms of overlap in children identified as at high risk of having a mental health condition. Knowledge of how these measures relate to each other could prove useful for comparisons across data-sets, given they are all commonly used. Hence, we aimed to determine the extent to which the SDQ, RCADS and short Mood and Feeling Questionnaire (sMFQ) identified the same children as at risk, defined as scoring above accepted cut-off points, and to determine the extent of correlation between these measures. We hypothesised that the SDQ-Emotional (SDQ-E) and RCADS-Total (RCADS-T), plus the RCADS-Depression (RACDS-D) and sMFQ, would have the greatest overlap. Likewise, we anticipated that the SDQ-Internalising (SDQ-I) and emotional subscales would be more strongly correlated with the RCADS-T, plus the RCADS-D and sMFQ scores, than the other subscales. Finally, in the child report, we expected that the school environment and social acceptance subscales of KIDSCREEN would be negatively correlated with all subscales except the SDQ-Prosocial behaviour subscale, which would show a positive correlation.

## Method

### Participants

A cross-sectional, follow-on survey was conducted in children aged 11–16 years and their parents, sampled from the participants in the Mental Health of Children and Young People (MHCYP) Survey Wave 2 follow-up that aimed to assess the mental health of children and young people in England.^19^ The COVID-19 pandemic prompted follow-up surveys^20^ of this sample, and the first (summer 2020) sought consent for contact to participate in mood and stress monitoring via a mobile app, MyJournE.^21^ A total of 889 children with their parents were approached between mid-October and November 2021 during partial school closures and lockdown in England, with twice weekly reminders to those who had not enrolled.

### Survey procedure

MyJournE is a secure and free app and e-platform co-designed with young people and researchers at King’s College London.^21^ Participants were enrolled in the study via email from which they were able to access and download the MyJournE app. Participant as well as parental e-consents were embedded within the mobile app and MyJournE e-platform, respectively. Participants were sign-posted to carefully selected health resources throughout the project, which included both participant material (e.g. participant information sheet) and the ‘support pages’ in the app itself. The survey took approximately 15 minutes to complete.

The authors assert that all procedures contributing to this work comply with the ethical standards of the relevant national and institutional committees on human experimentation and with the Helsinki Declaration of 1975, as revised in 2013. All procedures involving human participants/patients were approved by University of Cambridge Psychology Research Ethics Committee (PRE.2021.047).

### Measures

#### RCADS

MyJournE used the 11-item version of the RCADS.^10^ For children we selected a cut-off point of ≥7.5 for anxiety, ≥8.5 for depression and ≥12.5 for total score and for parents ≥5.5 for anxiety, ≥6.5 for depression and ≥10.5 for total score based on the recent UK validation study.^10^

#### SDQ

The self-report version was integrated into the app.^22^ We used the four-band categorisation of the SDQ scores.^23^ For total scores and sub-domains (except for prosocial behaviour where ‘low’ is the cut-off point), the ‘high’ category (reflecting top 10% of abnormal scores) was set as the cut-off point, which corresponds to a score of ≥18 for total difficulty (≥17 parent report), ≥6 for emotional problems (≥5 parent report), ≥5 for conduct problems (≥4 parent report), ≥7 for hyperactivity (≥8 parent report), ≥4 peer problem (≥4 parent report) and ≤5 for prosocial behaviour (≤6 parent report). Child-report SDQ cut-off points for internalising (emotional + peer problem) and externalising (hyperactivity + conduct problems) scales, which are not routinely used in clinical practice, were computed using the British Child and Adolescent Mental Health Survey 1999 data-set^24^ and were determined at the 90th percentile, which corresponded to scores of ≥8 and ≥10 for internalising and externalising scales, respectively.

#### sMFQ

The app incorporated the 13-item self-report version.^25^ We used the cut-off point set at a score of ≥12 based on a validation study in New Zealand conducted among a similar aged sample of adolescents.^26^ For the sMFQ parent report we used a cut-off point of ≥11 based on a validation study in a UK community sample.^27^

#### KIDSCREEN

The app included two domains of the child-report 52-item version: school environment, which reflected on the child’s well-being at school (six items), and social acceptance, that is, whether they were fearful of school and whether they were being bullied (three items). As there were no recommended cut-off points for either of these subscales, we used the 25th percentile, below which the HRQoL was considered poor as applied in a study that was conducted in six European countries including the UK.^28^ The score that corresponded to the 25th percentile for the school environment was 60 from a range of 23–100 and 87 for social acceptance from a range of 20–100.

### Statistical analysis

Statistical analysis was carried out using IBM SPSS 22.0 software on Windows. We calculated the raw scores for each measure, explored their distribution (mean and s.d.) and calculated the proportions scoring above cut-off points with 95% confidence intervals to allow comparison of estimates.

Differences between the app sample and the national sample for the SDQ and the app sample and European norms for KIDSCREEN measures were analysed using one-sample *t*-tests using the MHCYP follow-up 2021^20^ as the reference mean for the SDQ and the mean percentage scores from the European norm data^29^ for KIDSCREEN. Suitable UK norms for the RCADS and sMFQ were not available for comparison.

We constructed Venn diagrams to illustrate the overlaps among those identified as being at risk of mental health conditions because they scored above our selected cut-off points between conceptually similar scales and subscales of the SDQ, RCADS and sMFQ measures, and assessed chance-corrected agreement on at-risk caseness using the kappa statistic. Finally, we assessed Pearson’s correlations between all four measures.

## Results

Of the 899 children and parents approached, 231 children and 289 parents participated in our survey, giving response rates of 26% and 32%, respectively. The age of respondents ranged from 11 to 16 years (mean 13.92; s.d. = 1.74), and 53.3% were girls. Compared to the national sample, the MyJournE sample reported statistically significantly higher mean scores for total difficulty (*p* < 0.001), emotional problems (*p* < 0.001), conduct problems and hyperactivity (*p* = 0.002) and significantly lower mean prosocial behaviour scores (*p* = 0.002), indicating that we recruited a subsample of children with poorer mental health. Sub-analysis of the genders revealed a statistically significant elevation of emotional problems only among boys (*p* < 0.001), while girls showed a significant elevation of scores in all domains except peer problems and a significant lower mean of prosocial behaviour scores (*p* < 0.001) (Supplementary Table S1). In contrast, mean scores of school environment-related HRQoL in the app sample were significantly higher (*p* < 0.001) compared to European norms,^29^ indicating a better HRQoL (Supplementary Table S2).

Table 1 describes the distribution of child and parent scores from the SDQ, RACDS, sMFQ and child-report KIDSCREEN and the proportion scoring above cut-off point. Notably the number of children scoring above cut-off point for the SDQ total difficulty score (SDQ-T) (19.1% child report and 16.6% parent report) was lower than that for the RCADS-T (26.8% child report and 19% parent report). According to the SDQ subscales, emotional problems were the most commonly reported difficulty by both children and parents. The number of children scoring above the cut-off point on the SDQ-E was similar to the number scoring above the cut-off point on the RCADS-T for child report (26% and 26.8%, respectively), although it was higher for parent report (26% and 19%, respectively). Almost twice as many children scored themselves as being at risk of internalising disorders (29.9%) compared to externalising disorders (15.2%). The number of children scoring above the cut-off point on the sMFQ was higher than on the RCADS-D for both child report (19.9% and 14.7%, respectively) and parent report (12.1% and 8.7%, respectively).

**Table 1:** Proportion of children scoring above the cut-off point, at risk of mental health conditions, on the Strength and Difficulty Questionnaire (SDQ), Revised Children’s Anxiety and Depression Scale (RCADS), short Mood and Feeling Questionnaire (sMFQ) and KIDSCREEN and distribution of scores, by child and parent report

	Child report, at risk of mental health conditions^a^			Parent report, at risk of mental health conditions^b^		
	*n* (%)	Mean	s.d.	*n* (%)	Mean	s.d.
SDQ						
Total difficulty	44 (19.1)	11.4	6.8	48 (16.6)	9.4	6.7
Emotional problems	60 (26.0)	3.6	2.7	75 (26.0)	3.0	2.7
Conduct problems	18 (7.8)	1.6	1.8	27 (9.3)	1.3	1.5
Hyperactivity	50 (21.7)	4.1	2.7	21 (7.3)	3.3	2.6
Peer problems	40 (17.3)	1.9	1.8	59 (20.4)	1.9	2.0
Prosocial behaviour	27 (11.7)	7.6	1.8	70 (24.2)	7.8	2.0
Internalising scale	69 (29.9)	5.5	4.0	–	–	–
Externalising scale	35 (15.2)	5.8	3.9	–	–	–
RCADS						
Anxiety	57 (24.7)	5.3	4.3	58 (20.1)	3.7	3.4
Depression	34 (14.7)	4.5	3.6	25 (8.7)	2.9	2.8
Total (anxiety + depression)	62 (26.8)	9.8	7.5	55 (19.0)	6.6	5.8
sMFQ						
Depression	46 (19.9)	6.5	0.4	35 (12.1)	4.0	5.1
KIDSCREEN						
School environment	52 (22.5)	71.2	16.6	–	–	–
Social acceptance	47 (20.3)	90.4	13.9	–	–	–

Child report *N* = 231, parent report *N* = 289.

a. Cut-off point child report for the SDQ (scoring at and above ‘high’ corresponding to scores ≥18 for total difficulty, ≥6 for emotional problems, ≥5 for conduct problems, ≥7 for hyperactivity, ≥4 peer problem, ≤5 for prosocial behaviour (prosocial behaviour scores inversely and not included in total score), ≥8 for internalising and ≥10 for externalising subscales); for the RCADS (disregarding gender, ≥7.5 for anxiety, ≥8.5 for depression and ≥12.5 for total score); for the sMFQ (≥12) and for KIDSCREEN (scores <25th percentile corresponding to a score of 60.0 for school environment and 86.6 for social acceptance).

b. Cut-off point parent report for the SDQ (scoring at and above ‘high’ corresponding to scores ≥17 for total difficulty, ≥5 for emotional problems, ≥4 for conduct problems, ≥8 for hyperactivity, ≥4 peer problem and ≤6 for prosocial behaviour (prosocial behaviour scores inversely and not included in total score), for the RCADS (disregarding gender, ≥5.5 for anxiety, ≥6.5 for depression and ≥10.5 for total score); for the sMFQ (≥11).

**Fig. 1:**
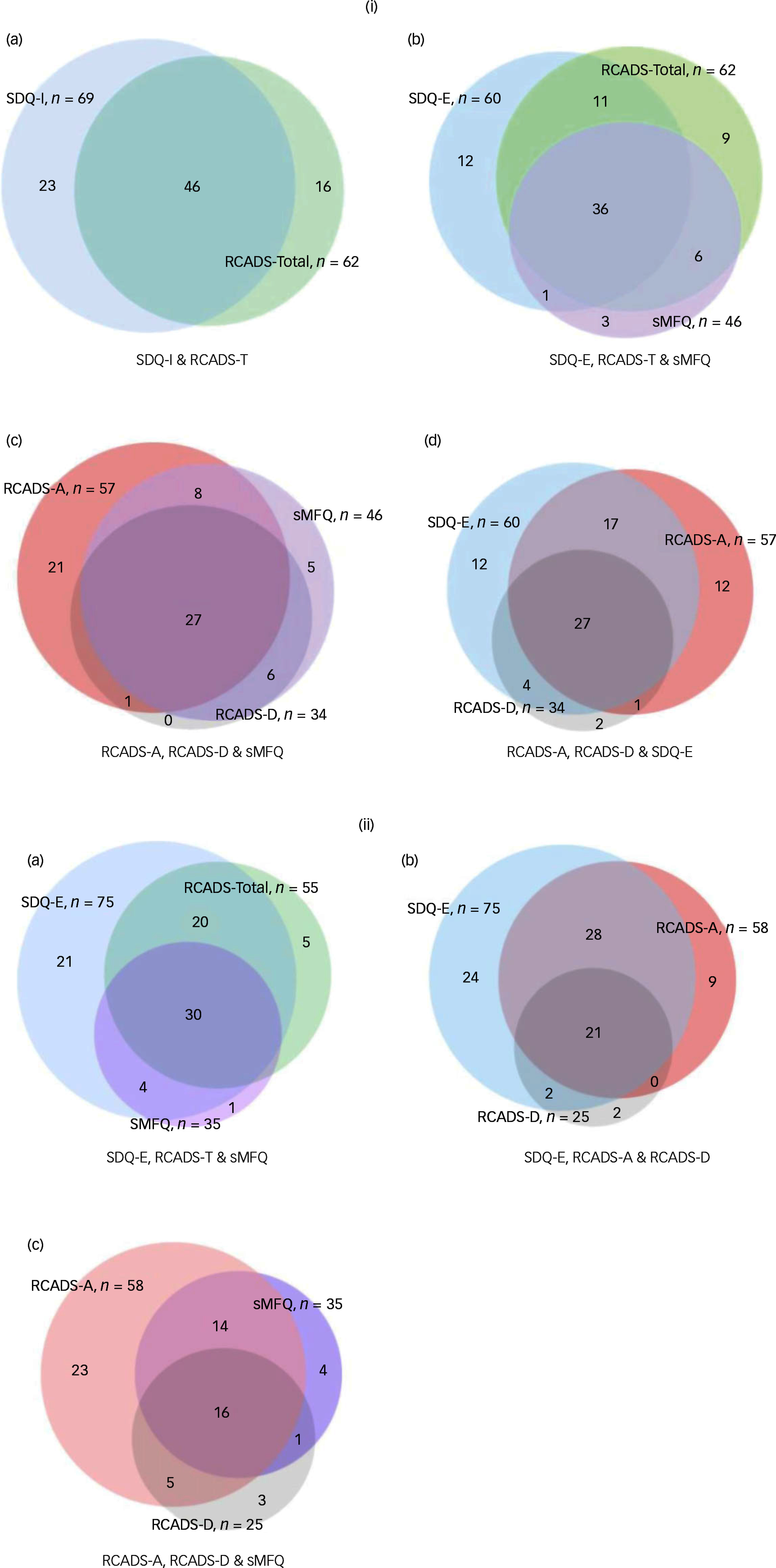
(i) Panel comparing the child self-report above cut-off points on (a) the Strength and Difficulty Questionnaire (SDQ)-Internalising (SDQ-I) and Revised Children’s19 Anxiety and Depression Scale (RCADS)-Total (RCADS-T); (b) SDQ-Emotional (SDQ-E), RCADS-T and short Mood and Feeling Questionnaire (sMFQ); (c) RCADS-Depression (RCADS-D), RCADS-Anxiety (RCADS-A) and sMFQ; (d) SDQ-E, RCADS-A and RCADS-D (representative, not to scale). *N* = 231. (ii) Panel comparing parent report above cut-off points on (a) the SDQ-E, RCADS-T and sMFQ; (b) SDQ-E, RCADS-A and RCADS-D; (c) RCADS-D, RCADS-A and sMFQ (representative, not to scale). *N* = 289.

Figure 1(i) provides a range of Venn diagrams to illustrate how these measures compared with each other in terms of the children scoring above the cut-off point. Figure 1(a) illustrates a moderate overlap (*n* = 46; 54.1%) of all children scoring above the cut-off point by the RCADS-T and SDQ-I scale. Figure 1(i)(b) illustrates how, despite a similar frequency of children scoring above the cut-off point, the RCADS-T (*n* = 62) and SDQ-E (*n* = 60) only identified 47 children in common with each other, with the RCADS-T ‘missing’ 13 children identified by the SDQ-E and similarly the SDQ-E ‘missing’ 15 children identified by the RCADS-T. Figure 1(i)(c) highlights that out of a total of 63 children who scored above the cut-off point on the RCADS-D, RCADS-Anxiety (RCADS-A) or sMFQ, 28 (44%) children were identified by both the RCADS-D and RCADS-A, reflecting the common comorbidity between these two conditions. The overlap of children scoring above the cut-off point between the SDQ-E and RCADS-A was greater than depression on the RCADS-D, which is not surprising as the SDQ-E comprises four questions about fears and worries and only one about sadness. In Fig. 1(i)(d), using the general scale, the SDQ-E ‘missed’ 15 children (20%) who scored above the cut-off point on the RCADS-A and RCADS-D. Conversely, the specific RCADS scales do not identify 12 children (16%) who scored above the cut-off point on the SDQ. Similarly, the sMFQ did not identify above the cut-off point 22 children (32%) that the RCADS-D did, whereas in the reverse comparison, the RCADS-D ‘missed’ five children scoring above the cut-off point (7%) which the sMFQ did (Fig. 1(i)c).

Figure 1(ii) provides a similar representation using Venn diagrams for parent report. Figure 1(ii)(a) highlights how the SDQ-E identifies nearly all children identified by the sMFQ, and the large proportion of those identified by the RCADS-T (91%). In Fig. 1(ii)(b), using the general scale, the SDQ-E ‘missed’ 11 children (12%) who scored above the cut-off point on the RCADS-A and RACDS-D. Conversely, neither of the RCADS subscales identify the 24 children (28%) who scored above the cut-off point on the SDQ-E. Figure 1(ii)(c) shows that the overlap in children scoring above the cut-off point between the sMFQ and RCADS-D is modest. Considering the total of 43 cases identified by both, the sMFQ ‘misses’ eight children (19%) scoring above the cut-off point on the RCADS-D, while the RCADS-D ‘misses’ 18 children (41%) that the sMFQ did.

Table 2 depicts correlations of RCADS and sMFQ scores with SDQ scores for both raters. Despite the modest overlap in children scoring above the cut-off point between measures, we observed moderate to high correlations across all measures. Predictably, the SDQ-E scores were significantly, strongly and positively correlated with measures of anxiety (RCADS-A) and depression (RCADS-D and sMFQ) and RCADS-T scores. Similarly, the child-report SDQ-I scores, which are a combination of both emotional and peer problems, demonstrated significant, positive and equally strong correlations with anxiety and depression measures, and these were not markedly different to the emotional subscale alone. Notably, the SDQ-T, which also incorporates attention and behavioural difficulties, demonstrated correlations that were only very slightly weaker than the emotional and internalising subscales, as indicated by the overlapping confidence intervals. Similarly, there were moderate and positive correlations of conduct and hyperactivity domains with the RCADS and sMFQ scales, implying either considerable comorbidity or conceptual overlap. As expected, prosocial behaviour scores showed significant but negative correlations with anxiety and depression scores.

**Table 2:** Pearson’s correlation of anxiety, depression and total scores (from the Revised Children’s Anxiety and Depression Scale (RCADS)) and depression scores (from the short Mood and Feeling Questionnaire (sMFQ)) with total and sub-domains of Strength and Difficulty Questionnaire (SDQ) scores

Child-report SDQ sub-domains	RCADS, child report						sMFQ, child report	
	Anxiety scores		Depression scores		Total scores		Depression scores	
	Correlation coefficient (*r*)	95% CI	Correlation coefficient (*r*)	95% CI	Correlation coefficient (*r*)	95% CI	Correlation coefficient (*r*)	95% CI
Total difficulty scores	0.70*	0.64–0.77	0.73*	0.66-0.79	0.76*	0.71–0.81	0.73*	0.68–0.79
Emotional problem scores	0.80*	0.75–0.85	0.77*	0.71–0.82	0.84*	0.79–0.87	0.77*	0.71–0.82
Conduct problem scores	0.31*	0.19–0.42	0.36*	0.07–0.48	0.35*	0.23–0.47	0.38*	0.24–0.51
Hyperactivity problem scores	0.41*	0.29–0.52	0.44*	0.33–0.54	0.45*	0.35–0.55	0.48*	0.37–0.57
Peer problems	0.52*	0.40–0.64	0.56*	0.45–0.65	0.58*	0.45–0.68	0.53*	0.43–0.63
Prosocial behaviour scores	−0.15**	−0.29 to −0.01	−0.24*	−0.36 to −0.10	−0.2**	−0.34 to −0.07	−0.21**	−0.33 to −0.82
Internalising problems	0.78*	0.72–0.84	0.78*	0.72–0.83	0.83*	0.78–0.87	0.76*	0.69–0.82

Parent-report SDQ sub-domains	RCADS, parent report						sMFQ, parent report	
	Anxiety scores		Depression scores		Total scores		Depression scores	
	Correlation coefficient (*r*)	95% CI	Correlation coefficient (*r*)	95% CI	Correlation coefficient (*r*)	95% CI	Correlation coefficient (*r*)	95% CI
Total difficulty scores	0.69*	0.63–0.75	0.67*	0.61–0.73	0.74*	0.68–0.78	0.77*	0.71–0.81
Emotional problem scores	0.77*	0.73–0.82	0.70*	0.64–0.76	0.80*	0.75–0.84	0.72*	0.66–0.77
Conduct problem scores	0.31*	0.20–0.41	0.41*	0.31–0.51	0.38*	0.28–0.48	0.54*	0.46–0.62
Hyperactivity problem scores	0.38*	0.28–0.46	0.34*	0.23–0.44	0.39*	0.29–0.48	0.47*	0.38–0.56
Peer problems	0.563*	0.48–0.64	0.56*	0.48–0.64	0.61*	0.53–0.68	0.57*	0.49–0.65
Prosocial behaviour scores	−0.34*	−0.44 to −0.23	−0.40*	−0.49 to −0.30	−0.4*	−0.49 to −0.29	−0.45*	−0.54 to −0.36
sMFQ	0.73*	0.67–0.78	0.77*	0.72–0.82	0.83*	0.76–0.84	0.76*	0.69–0.82

Significant at **p < 0.05 level; *p < 0.001 level.

Considering correlations between psychopathology scales and the KIDSCREEN scale, we found moderate to strong correlations between total and subscale scores of all measures (SDQ, RCADS and sMFQ) with the school environment: positive for prosocial behaviour and negative for all the others. Although the social acceptance subscale showed a similar pattern, correlations with prosocial behaviour and correlations with the conduct and hyperactivity subscales were surprisingly small and not statistically significant (Table 3).

**Table 3:** Correlation between school environment and social acceptance (KIDSCREEN scores) with the Strength and Difficulty Questionnaire (SDQ), Revised Children’s Anxiety and Depression Scale (RCADS) and short Mood and Feeling Questionnaire (sMFQ), child report

Parameter	School environment		Social acceptance	
	Correlation coefficient (*r*)	95% CI	Correlation coefficient (*r*)	95% CI
Total difficulty (SDQ)	−0.65*	−0.73 to −0.56	−0.4*	−0.5 to −0.2
Emotional problems (SDQ)	−0.55*	−0.64 to −0.44	−0.4*	−0.5 to −0.3
Conduct problems (SDQ)	−0.47*	−0.57 to −0.35	−0.1	−0.2 to −0.05
Hyperactivity (SDQ)	−0.51*	−0.61 to −0.41	−0.1	−0.2 to −0.02
Peer problems (SDQ)	−0.41*	−0.51 to −0.28	−0.5*	−0.6 to −0.4
Prosocial behaviour (SDQ)	0.33*	0.19 to 0.45	0.01	−0.10 to 0.14
Internalising problems (SDQ)	−0.55*	−0.65 to −0.45	−0.5*	−0.6 to −0.4
Anxiety (RCADS)	−0.50*	−6.0 to −0.38	−0.5*	−0.6 to −0.4
Depression (RCADS)	−0.61*	−0.68 to −0.52	−0.4*	−0.5 to −0.3
Total (RCADS)	−0.58*	−0.67 to −0.48	−0.5*	−0.6 to −0.4
Depression (sMFQ)	−0.61*	−0.68 to 0.51	−0.4*	−0.5 to −0.3

*Significant at *p* < 0.001 level.

Kappa statistics was performed to determine the chance-corrected agreement between scales/subscales measuring similar psychological constructs and ranged between 0.52 and 0.77 (*p* < 0.001), showing moderate to substantial strength of agreement (Table 4).

**Table 4:** Chance-corrected agreement between scales

Scales	Kappa	95% CI	Strength of agreement
Child-report scales			
RCADS-A and SDQ-E	0.66	0.54–0.78	Substantial
RCADS-T and SDQ-I	0.59	0.47–0.71	Moderate
RCADS-T and SDQ-E	0.69	0.50–0.81	Substantial
RCADS-T and sMFQ	0.71	0.61–0.81	Substantial
RCADS-D and sMFQ	0.77	0.65–0.89	Substantial
SDQ-E and sMFQ	0.61	0.49–0.73	Substantial
Parent-report scales			
RCADS-A and SDQ-E	0.66	0.56–0.76	Substantial
RCADS-T and SDQ-E	0.70	0.60–0.80	Substantial
RCADS-T and sMFQ	0.61	0.49–0.73	Substantial
RCADS-D and sMFQ	0.52	0.36–0.68	Moderate
SDQ-E and sMFQ	0.54	0.42–0.66	Moderate

RCADS, Revised Children’s Anxiety and Depression Scale; RCADS-A, RCADS-Anxiety; SDQ, Strength and Difficulty Questionnaire; SDQ-E, SDQ-Emotional; RCADS-T, RCADS-Total; SDQ-I, SDQ-Internalising; sMFQ, short Mood and Feeling Questionnaire; RCADS-D, RCADS-Depression.

## Discussion

We aimed to explore the extent to which four common measures of child psychopathology related to each other, particularly in identifying the same children above the recommended cut-off points. We found moderate to high correlations between measures as expected, but surprisingly, relatively modest overlap in the children identified as at risk of mental health conditions even between conceptually similar measures. Our findings also revealed surprisingly low correlations among hyperactivity, conduct problems and prosocial behaviour with social acceptance.

We anticipated that the SDQ would identify a greater number of children at risk of mental health difficulty than the RCADS or sMFQ, given that it assesses a broader range of problems. However, our study revealed contrasting findings. Goodman et al^30^ cautioned against the use of the five-factor structure of the SDQ in low-risk community samples owing to its poor discriminant validity between emotional and peer problem domains. Instead, a more conservative approach of using internalising and externalising subscales was recommended. Our findings fit this recommendation as the SDQ-T cut-off point identified a lower number of children than the RCADS-T, whereas the SDQ-E or SDQ-I subscale identified similar numbers.

Our study reported very strong, positive correlations between the SDQ-E, SDQ-I and SDQ-T scores with RCADS and sMFQ scores. These findings suggest the usefulness of a general questionnaire such as the SDQ in indicating the presence of potential anxiety or depressive disorder, as well as the advantage of a broader range of questions that could denote the presence of disorders in other domains.^31^

Although both the SDQ-I and RCADS-T identified similar proportions of young people as possible cases, we observed only modest overlap *between* the two internalising scales despite their conceptual similarity. Differences possibly emerge from the structured components; while both scales are similarly structured to measure anxiety, the RCADS consists of an exclusive subcomponent to measure depression while the SDQ-I contains one question about sadness and combines the emotional and peer relationship problem subscales. Furthermore, the peer problem items, which include questions that assess preference for solitude, lack of friends, being unpopular with other children, better relationships with adults than children and being bullied, do not exclusively map onto internalising difficulties.

Our child-report sample reported higher levels of internalising problems than externalising problems, which is consistent with the National Survey Programme (Mental Health of Children and Adolescents of Great Britain and MHCYP surveys) that indicated a significant increase in emotional disorders among 5–15-year-olds between 1999, 2004 and 2017, while behavioural and hyperactivity disorders remained broadly stable.^19^ The proportion of young people reporting difficulties in focusing attention, however, is markedly higher than these earlier national surveys of psychiatric disorders would predict. These findings add to contradictory reports about the impact of the COVID-19 pandemic and its resultant restrictions on these same scales.^32^

In our sample, the sMFQ identified more children above the cut-off point compared to the RCADS-D. Evidence shows that the two additional symptom impact items related to distress and interference at school contribute to improved accuracy of the 11-item RCADS^10^ and is consistent with a plethora of previous research that highlights the superiority of including impact items over symptom items alone when estimating the prevalence of clinically impairing mental health problems in young people.^33–35^ In addition, Radez et al^10^ demonstrated that for the 11-item RCADS, adolescent report combined with parent report generated the most accurate results, as have other similar studies of multiple versus single informants.^36–38^ The RCADS is highly sensitive to gender differences,^39^ while the sMFQ has been shown to measure depression equivalently in males and females.^40^ We chose to use a common cut-off disregarding gender in our study for the RCADS, which may have contributed to its underperformance, but it remains interesting to consider why the RCADS requires different thresholds for girls and boys when few other psychopathology measures do. As expected, there was a greater overlap between the RCADS-D and sMFQ than between the RCADS-D and RCADS-A. This was also reported by Radez et al,^10^ who established a highly convergent validity between these depression scales with moderate to high correlation scores. These authors also detected moderate correlations between sMFQ and RCADS-A scores, reflective of the known common comorbidity between anxiety and depressive symptoms,^19^ which was also evident in our study.

Our study replicates results from prior work establishing the inverse associations between adverse school environment and anxiety, depression and internalising and externalising problems in children^41,42^; a Finnish population-based longitudinal study revealed the close association of a poor classroom climate with internalising behaviour.^43^ Similarly, social acceptance displayed inverse associations with the SDQ-I, RCADS-A, RCADS-D and sMFQ, which is consistent with previous studies.^44,45^ However, contrary to our hypothesis, social acceptance neither demonstrated a significant negative correlation with behavioural problems, nor a significant positive correlation with prosocial behaviour. The three items of the social acceptance domain of the KIDSCREEN questionnaire focus on identifying children who are victims of bullying. Evidence suggests that victims of bullying (implying low social acceptance) are at greater risk of suffering from internalising symptoms, while behavioural problems are more common among bullies.^46–48^ A good classroom climate with a positive student–teacher relationship may have had a cushioning effect against bullying,^49^ which may have not affected prosocial behaviour adversely. Alternatively, the unusual social and school circumstances during data collection, when schools were partially open but with social isolation for those groups exposed to infection, may have affected both the school experience and reporting.

Our findings emphasise that no measure is perfect for all situations. This issue has led to a plethora of scales to measure the same construct. One possible solution is the use of computer-adapted testing, which pools a large collection of items from various instruments calibrated using item response theory to generate short personalised assessments.^50^ Instead of studying whole instruments separately, these models enable identifying useful items and evaluating the extent of overlap between instruments and the best set of items for specific assessment purposes.

### Strengths and limitations

Our study benefitted from a moderately large sample of young people who completed these commonly used mental health scales simultaneously. The sample who took part was a self-selecting group from within another study and differed from the wider study group, indicating poorer mental health. This selection bias towards those with higher psychopathology and a tendency to score above cut-off points in screening questionnaires may relate to the primary purpose of the study, which invited people to monitor their mood and mental health over time. However, in relation to our research question, which was an in-group comparison, generalisability to the general population is not essential. Indeed, the group more closely represents those who may seek clinical help, which increases the relevance of our findings to the clinical population and is considered a strength of the study. Inevitably there are some methodological limitations. First, the MFQ and RCADS applied were both short versions and evidence of their psychometric properties and measures of diagnostic accuracy are scarcer than for the longer, original versions. However, the shorter version of the RCADS has been recommended when brevity is required, as is often the case in clinical practice or large surveys.^5^ Second, the lack of impact scores on the SDQ and RCADS restricted taking into account the overall distress and level of functional impairment when reaching a decision on cut-off points. The choice of cut-off points for each scale will have influenced the overlap, but we used those most strongly endorsed for each measure.

These questionnaires, particularly the SDQ and RCADS, are routinely used in clinical child and adolescent mental health services (CAMHS) settings in the UK, at triage and initial assessment. Despite strong correlations between these commonly used measures, there is surprisingly modest overlap between children identified above the cut-off point by child or parent report. Taken at the individual child level, a clinician cannot be wholly confident that if a child scores below the cut-off on a screening test, by child or parent report, that they will score below the cut-off in other screening tests correlating to similar psychopathologies. This highlights the risk of using a screening test to ‘rule out’ potential psychopathology at triage without further assessment. Screening tests should not be used diagnostically, but are best used in conjunction with broad assessment and contain valuable information regardless of whether the cut-off point is reached. Researchers, clinicians and policymakers must consider the relative strengths and weaknesses of particular measures in relation to their intended purpose.

## Supporting information

Nazeer et al. supplementary material 1 Nazeer et al. supplementary material

Nazeer et al. supplementary material 2 Nazeer et al. supplementary material

## Data Availability

Data is available on request from the corresponding author.

## Supplementary material

The supplementary material for this article can be found at https://doi.org/10.1192/bjp.2025.5
