# Supplementary material for: The extent to which child- and parent-report Revised Children’s Anxiety and Depression Scale, short Mood and Feeling Questionnaire, Strength and Difficulty Questionnaire and child-report KIDSCREEN identify the same young people as at risk of mental health conditions
Source: Br J Psychiatry. 2025 Mar 26;228(3):195–204. doi: 10.1192/bjp.2025.5 (PMC7617555; doi:10.1192/bjp.2025.5)
Supplement: Nazeer et al. supplementary material 1 — Nazeer et al. supplementary material [file S0007125025000054sup001.docx]

**Supplementary Tables**

Table: S1 Comparison of the mean SDQ scores between MyJournE App data sample and the National sample(Newlove-Delgado et al., 2021)

|  | A: MyJournE App data sample | | | B: National sample (from 2021 follow-up survey) | | | Difference  (A-B) | | | t statistics | Statistical significance  (p) |
| --- | --- | --- | --- | --- | --- | --- | --- | --- | --- | --- | --- |
|  | Mean | ͣ LCL | ᵇUCL | Mean | LCL | UCL | Mean | LCL | UCL |  |  |
| **All** | | | | | | | | | | | |
| Mean total difficulty score | 11.4 | 10.5 | 12.3 | 9.6 | 9.2 | 10.0 | 1.8 | 0.9 | 2.6 | 3.9 | <0.001 |
| Mean emotional problem score | 3.6 | 3.3 | 4.0 | 2.5 | 2.3 | 2.6 | 1.2 | 0.8 | 1.5 | 6.5 | <0.001 |
| Mean conduct problem score | 1.6 | 1.5 | 1.9 | 1.4 | 1.3 | 1.5 | 0.3 | 0.05 | 0.5 | 2.3 | 0.002 |
| Mean hyperactivity problem score | 4.1 | 3.8 | 4.5 | 3.6 | 3.5 | 3.8 | 0.5 | 0.2 | 0.9 | 3.1 | 0.002 |
| Mean peer problem score | 1.9 | 1.6 | 2.1 | 2.1 | 1.9 | 2.2 | -0.3 | -0.4 | 0.03 | -1.8 | 0.09 |
| Mean prosocial behaviour score | 7.6 | 7.4 | 7.9 | 8.0 | 7.9 | 8.1 | -0.4 | -0.6 | -0.1 | -3.1 | 0.002 |
| **Boys** | | | | | | | | | | | |
| Mean total difficulty score | 9.5 | 8.3 | 10.6 | 9.6 | 9.1 | 10.2 | -0.07 | -1.2 | 1.1 | -1.2 | 0.908 |
| Mean emotional problem score | 3.6 | 3.1 | 4.1 | 2.0 | 1.8 | 2.2 | 1.6 | 1.1 | 2.2 | 6.1 | <0.001 |
| Mean conduct problem score | 1.7 | 1.3 | 2.0 | 1.5 | 1.3 | 1.6 | 0.20 | -0.12 | 0.52 | 1.2 | 0.223 |
| Mean hyperactivity problem score | 4.0 | 3.5 | 4.5 | 4.0 | 3.8 | 4.2 | 0.04 | -0.47 | 0.55 | 0.14 | 0.886 |
| Mean peer problem score | 1.4 | 1.8 | 2.1 | 2.0 | 2.1 | 2.3 | -0.03 | -0.35 | 0.29 | -0.19 | 0.846 |
| Mean prosocial behaviour score | 7.6 | 7.3 | 7.9 | 7.7 | 7.5 | 7.9 | -0.08 | -0.42 | 0.26 | -0.478 | 0.634 |
| **Girls** | | | | | | | | | | | |
| Mean total difficulty score | 12.9 | 11.7 | 14.2 | 9.5 | 9.0 | 10.0 | 3.5 | 2.2 | 4.7 | 5.5 | <0.001 |
| Mean emotional problem score | 3.6 | 3.2 | 4.1 | 3.0 | 2.8 | 3.2 | 0.68 | 0.2 | 1.2 | 2.8 | 0.005 |
| Mean conduct problem score | 1.6 | 1.3 | 1.9 | 1.3 | 1.2 | 1.4 | 0.36 | 0.02 | 0.69 | 2.1 | 0.036 |
| Mean hyperactivity problem score | 4.2 | 3.7 | 4.7 | 3.2 | 3.1 | 3.4 | 1.04 | 0.57 | 1.5 | 4.4 | <0.001 |
| Mean peer problem score | 1.9 | 1.6 | 2.3 | 2.0 | 1.8 | 2.1 | -0.03 | -0.34 | 0.29 | -0.19 | 0.846 |
| Mean prosocial behaviour score | 7.6 | 7.3 | 7.9 | 8.3 | 8.1 | 8.4 | -0.66 | -0.33 | -0.99 | -4.00 | <0.001 |

*ͣ LCL= lower confidence limit*

*ᵇUCL= upper confidence limit*

Table: S2 Comparison of KIDSCREEN scores between MyJournE App sample and European norms(Ravens-Sieberer et al., 2006)

| Parameter | MyJournE App sample | | European Norms | | Mean differences (95% CI) | t statistics | Significance (p) |
| --- | --- | --- | --- | --- | --- | --- | --- |
|  | Mean S score | ͣ SD | Mean S Score | SD |  |  |  |
| School environment | 71.2 | 16.6 | 66.39 | 20.90 | 4.78 (2.6-6.9) | 4.30 | <0.001 |
| Social acceptance | 90.4 | 13.9 | 88.90 | 16.53 | 1.5 (-0.3-3.3) | 1.6 | 0.09 |

*ͣ SD=Standard Deviation*
