# Supplementary material for: The extent to which child- and parent-report Revised Children’s Anxiety and Depression Scale, short Mood and Feeling Questionnaire, Strength and Difficulty Questionnaire and child-report KIDSCREEN identify the same young people as at risk of mental health conditions
Source: Br J Psychiatry. 2025 Mar 26;228(3):195–204. doi: 10.1192/bjp.2025.5 (PMC7617555; doi:10.1192/bjp.2025.5)
Supplement: Nazeer et al. supplementary material 2 — Nazeer et al. supplementary material [file S0007125025000054sup002.docx]

**Supplementary Text**

**The Revised Children’s Anxiety and Depression Scale (RCADS)** is composed of six subscales: Separation Anxiety Disorder, Social Phobia, Generalized Anxiety Disorder, Obsessive-Compulsive Disorder, Panic Disorder, and Major Depressive Disorder. These are scored across a 4-point Likert scale from 0-3 corresponding to “never”, “sometimes”, “often”, and “always”. Psychometric evaluation of RCADS-11 item scale among clinic and community samples of adolescents aged 11 to17 years in England revealed good diagnostic accuracy (Area Under the Curve (AUC) of 0.82) plus sensitivity and specificity values of 0.80 and 0.71 at a cut point of 12.5 respectively, disregarding gender. Omega coefficients reflected high internal consistency in both the clinic-referred (0.73-0.88) and community (0.87-0.94) samples (1). Moreover, RCADS has demonstrated its ability to detect changes following participation in short and long mental health interventions in children (2,3) and in detecting individual-level symptom improvement at clinical services (4).

**The Moods and Feelings Questionnaire (MFQ)** assesses the presence of affective and cognitive symptoms of depression with items rated on a 3-point Likert about experiences over the course of the last two weeks as being ‘true’, ‘sometimes true’, or ‘not true. The original MFQ has good diagnostic accuracy (AUC=0.82), a high internal consistency (α=0.94) and a high test-retest reliability (ICC=0.78) (5). The shorter 13-item version (sMFQ) showed similarly promising psychometric attributes across cultures and settings(6-9). The MFQ provides a valid and reliable option to monitor changes in depression in short cognitive behavioural interventions (10,11). The original authors recommend that cut points be decided by the users related to their circumstance (12).

**The Strengths and Difficulties Questionnaire (SDQ)** is composed of statements which the respondent identifies as either ‘Not True’, ‘Somewhat True’, or ‘Certainly True’ scored respectively 0, 1 and 2 with regards to their experience in the last 6 months. Half the statements are phrased positively, and reverse scored so that a high score indicates greater difficulty. Items of the first four subscales are then summed up to produce a “total difficulty score” ranging from 0-40. An impact supplement component assesse the extent of distress and impairment caused by any identified problem, which improves the accuracy of detection of disorders in individual children

(13).In addition, an externalising score can be created by summing scores from conduct and hyperactivity scales, while an internalising score is calculated by combining the emotional and peer problem scales. For public health and epidemiological uses, these scale function better as amalgamated scales, whereas the four-factor structure of the scale is of greater value in assessing high-risk samples (14).

The SDQ has strongly established psychometric properties determined in a nationwide epidemiological sample in the UK (15) as well as across diverse cultures around the globe; Germany (16), Netherlands (17), France (18), Australia (19), South Africa (20), Japan (21) and China (22). Being a broad measure, its utility to evaluate clinical effectiveness in specialist health care appeared less satisfactory compared to other measures of narrowed focus, but allows comparison across a wider range of difficulties than most focused measures (23). The SDQ has been used in several cluster RCTs in Ireland to successfully detect changes following short parent and co-parent interventions (24,25), as well as a cluster RCT teaching classroom management (26). For children aged 4-17 years a newer 4-band categorisation of scores has been validated identifying cut-points for all subscales across all respondents in the UK (27).

**KIDSCREEN** is applicable to healthy and chronically ill children and young people aged 8 to 18 years and is available in long (a 52-item and 27-item) as well as short (10-item) versions (28). Although developed as a self-reported measure it is also available as a parent or proxy measure. The 52-item version measures 10- health related quality of life (HRQoL) dimensions: Physical, Psychological Well- being, Moods and Emotions, Self- Perception, Autonomy, Parent Relations and Home Life, Social Support and Peers, School Environment, Social Acceptance (Bullying), and Financial Resources. The 27-item version amalgamates several of these components, excluding bullying, to create a 5-dimension format from which 10-items are derived to develop the shortest form. The responses are recorded on a Likert scale of 5 categories. Summation of scores of all items produces the dimension score which are transformed in to values between 0 to 100 where higher values signify better HRQoL. T scores are available for stratified by age, gender and socio-economic status (29). The 52-item KIDSCREEN has demonstrated acceptable levels of reliability in all 13 European countries that simultaneously developed the scale; Cronbach’s alpha values ranged from 0.76 -0.89 across all 10 dimensions (30). The same study established satisfactory levels of convergent and discriminant validity. There has been demonstrable evidence of its change to sensitivity in a population based longitudinal Spanish study (31) and the 11-year follow up of the renowned German longitudinal study (BELLA study; 32). Furthermore, the KIDSCREEN-10 has been endorsed by the International Consortium for Health Outcomes Measurement as a standardized global measure of well-being in children and adolescents(33).

**References**

1. Radez J, Waite P, Chorpita B, Creswell C, Orchard F, Percy R, et al. Using the 11-item Version of the RCADS to Identify Anxiety and Depressive Disorders in Adolescents. Res Child Adolesc Psychopathol. 2021 Sep 1;49(9):1241–57.

2. Stallard P, Skryabina E, Taylor G, Phillips R, Daniels H, Anderson R, et al. Classroom-based cognitive behaviour therapy ( FRIENDS ): a cluster randomised controlled trial to Prevent Anxiety in Children through Education in Schools ( PACES ). The Lancet Psychiatry [Internet]. 2014;1(3):185–92. Available from: http://dx.doi.org/10.1016/S2215-0366(14)70244-5

3. Kösters MP, Chinapaw MJM, Zwaanswijk M, Wal MF Van Der, Koot HM. Structure , reliability , and validity of the revised child anxiety and depression scale ( RCADS ) in a multi-ethnic urban sample of Dutch children. BMC Psychiatry [Internet]. 2015;1–8. Available from: http://dx.doi.org/10.1186/s12888-015-0509-7

4. Edbrooke-childs J, Wolpert M, Zamperoni V, Napoleone E, Bear H. Evaluation of reliable improvement rates in depression and anxiety at the end of treatment in adolescents. 2018;250–5.

5. Wood A, Kroll L, Moore A, Harrington R. Properties of the Mood and Feelings Questionnaire in Adolescent Psychiatric Outpatients : A Research Note. 1994;(September).

6. Messer S, Ephen Messer S, Messer SC, Ango A, Mrcp LD, Jane E, et al. DEVELOPMENT OF A SHORT QUESTIONNAIRE FOR USE IN EPIDEMIOLOGICAL STUDIES OF DEPRESSION IN CHILDREN AND ADOLESCENT... Related papers CHILDHOOD DEPRESSION AND AGGRESSION: A COVARIANCE ST RUCT URE ANALYSIS St ephen Messer DEVELOPME NT OF A SHORT QUEST IONNAIR. Int J Methods Psychiatr Res. 1995;5:25–262.

7. Thabrew H, Stasiak K, Bavin LM, Frampton C, Merry S. Validation of the Mood and Feelings Questionnaire (MFQ) and Short Mood and Feelings Questionnaire (SMFQ) in New Zealand help-seeking adolescents. Int J Methods Psychiatr Res. 2018 Sep 1;27(3).

8. Lerthattasilp T, Tapanadechopone P, Butrdeewong P. Validity and Reliability of the Thai Version of the Short Mood and Feelings Questionnaire. 2020;(December 2017):48–51.

9. Id J, Ivarsson T, Andersson M, Bergman H. Screening efficiency of the Mood and Feelings Questionnaire ( MFQ ) and Short Mood and Feelings Questionnaire ( SMFQ ) in Swedish help seeking outpatients. 2020;59.

10. Jensen TK, Holt T, Ormhaug SM, Egeland K, Granly L, Hoaas LC, et al. A Randomized Effectiveness Study Comparing Trauma-Focused Cognitive Behavioral Therapy With Therapy as Usual for Youth RANDOMIZED CLINICAL TRIALS A Randomized Effectiveness Study Comparing Trauma-Focused Cognitive Behavioral Therapy With Therapy as Usual . 2014;4416.

11. Wright B, Tindall L, Littlewood E, Allgar V, Abeles P, Trépel D, et al. Computerised cognitive – behavioural therapy for depression in adolescents : feasibility results and 4-month outcomes of a UK randomised controlled trial. 2017;

12. Angold A, Sharp EA, Daviss A, Burleson W, Nadine A, David A. Mood and Feeling Questionnaire. 2006;47(9).

13. Goodman R, Ford T, Simmons H, Gatward R, Meltzer H. Using the Strengths and Difficulties Questionnaire ( SDQ ) to screen for child psychiatric disorders in a community sample. Br J Psychiatry. 2000;177:534–9.

14. Goodman A, Lamping DL, Ploubidis GB. When to use broader internalising and externalising subscales instead of the hypothesised five subscales on the strengths and difficulties questionnaire (SDQ): Data from british parents, teachers and children. J Abnorm Child Psychol. 2010 Nov;38(8):1179–91.

15. Goodman R. Psychometric Properties of the Strengths and Difficulties Questionnaire. J Am Acad Child Adolesc Psychiatry [Internet]. 2001 Nov 1;40(11):1337–45. Available from: https://doi.org/10.1097/00004583-200111000-00015

16. Woerner W, Background A. Normative data and scale properties of the German parent SDQ. 2004;10.

17. Muris P. The Strengths and Difficulties Questionnaire ( SDQ ) Further evidence for its reliability and validity in a community sample of Dutch children and adolescents. Eur Child Adolesc Psychiatry. 2003;8:1–8.

18. Shojaei T, Wazana A, Pitrou I, Kovess V. The strengths and difficulties questionnaire : validation study in French school-aged children and cross-cultural comparisons. Soc Psychiatry Psychiatr Epidemiol. 2009;44:740–7.

19. Mellor D. Furthering the Use of the Strengths and Difficulties Questionnaire_ Reliability With Younger Child Respondents_.pdf. Am Psychol Assoc. 2004;16(4):396–401.

20. Vries PJ De, Davids EL, Mathews C, Aarø LE. Measuring adolescent mental health around the globe : psychometric properties of the self-report Strengths and Difficulties Questionnaire in South Africa , and comparison with UK , Australian and Chinese data. Epidemiol Psychiatr Sci. 2018;27:369–80.

21. Moriwaki A, Kamio Y. Normative data and psychometric properties of the strengths and difficulties questionnaire among Japanese school-aged children. Child Adolesc Psychiatry Ment Health. 2014;8(1):1–12.

22. Du Y, Kou J, Coghill D. Child and Adolescent Psychiatry and The validity , reliability and normative scores of the parent , teacher and self report versions of the Strengths and Difficulties Questionnaire in China. Child Adolesc Psychiatry Ment Health. 2008;15:1–15.

23. Lee W, Jones L, Goodman R, Heyman I. Broad outcome measures may underestimate effectiveness: An instrument comparison study. Child Adolesc Ment Health. 2005 Sep;10(3):143–4.

24. Keating A, Sharry J, Murphy M, Rooney B, Carr A. An evaluation of the Parents Plus – Parenting When Separated programme. Clin Child Psychol Psychiatry [Internet]. 2015 Apr 24;21(2):240–54. Available from: https://doi.org/10.1177/1359104515581717

25. Nitsch E, Hannon G, Rickard E, Houghton S, Sharry J. Positive parenting : a randomised controlled trial evaluation of the Parents Plus Adolescent Programme in schools. Child Adolesc Psychiatry Ment Health. 2015;1–12.

26. Ford T, Hayes R, Byford S, Edwards V, Fletcher M, Logan S, et al. The effectiveness and cost-effectiveness of the Incredible Years ® Teacher Classroom Management programme in primary school children: Results of the STARS cluster randomised controlled trial. Psychol Med. 2019;49(5):828–42.

27. Goodman R, Meltzer H, Bailey V. Scoring the Strengths & Difficulties Questionnaire for age 4-17 or 18+. 2016.

28. Ravens-Sieberer U, Gosch A, Abel T, Auquier P, Bellach BM, Bruil J, et al. Quality of life in children and adolescents: a European public health perspective. Soz Praventivmed. 2001;46(5):294–302.

29. Ravens-Sieberer U, Gosch A, Erhart M, Von Rueden U, Nickel J, Kurth B-M, et al. The KIDSCREEN questionnaires Quality of life questionnaires for children and adolescents-Handbook-THE KIDSCREEN GROUP EUROPE. 2006.

30. Ravens-sieberer U, Gosch A, Rajmil L, Erhart M, Bruil J, Power M, et al. The KIDSCREEN-52 Quality of Life Measure for Children and Adolescents : Psychometric Results from a Cross-Cultural Survey in 13 European Countries. Value Heal [Internet]. 2008;11(4):645–58. Available from: http://dx.doi.org/10.1111/j.1524-4733.2007.00291.x

31. Æ JMVÆMEÆMH, Rajmil SBÆJAÆL. Changes in health-related quality of life ( HRQoL ) in a population-based sample of children and adolescents after 3 years of follow-up. Qual Life Res. 2008;1207–15.

32. Otto C, Reiss F, Voss C, Wüstner A, Katrin A, Hölling H, et al. Mental health and well ‑ being from childhood to adulthood : design , methods and results of the 11 ‑ year follow ‑ up of the BELLA study. Eur Child Adolesc Psychiatry [Internet]. 2021;30(10):1559–77. Available from: https://doi.org/10.1007/s00787-020-01630-4

33. Krause KR, Chung S, Adewuya AO, Albano AM, Babins-wagner R, Birkinshaw L, et al. International consensus on a standard set of outcome measures for child and youth anxiety , depression , obsessive- compulsive disorder , and post-traumatic stress disorder. Lancet Psychiatry. 2021;8(January).
