# Supplementary material for: The extent to which child and parent report RCADS, sMFQ, SDQ and child report KIDSCREEN identify the same young people as at risk of mental health conditions
Source: Br J Psychiatry. Author manuscript; Available in PMC 2026 Mar 1. (PMC7617555; doi:10.1192/bjp.2025.5)
Supplement: Supplementary file 1 [file EMS202776-supplement-Supplementary_Materials.docx]

**Supplementary Text** 

**The Revised Children’s Anxiety and Depression Scale (RCADS)** is composed of six subscales: Separation Anxiety Disorder, Social Phobia, Generalized Anxiety Disorder, Obsessive-Compulsive Disorder, Panic Disorder, and Major Depressive Disorder. These are scored across a 4-point Likert scale from 0-3 corresponding to “never”, “sometimes”, “often”, and “always”. Psychometric evaluation of RCADS-11 item scale among clinic and community samples of adolescents aged 11 to17 years in England revealed good diagnostic accuracy (Area Under the Curve (AUC) of 0.82) plus sensitivity and specificity values of 0.80 and 0.71 at a cut point of 12.5 respectively, disregarding gender. Omega coefficients reflected high internal consistency in both the clinic-referred (0.73-0.88) and community (0.87-0.94) samples (1). Moreover, RCADS has demonstrated its ability to detect changes following participation in short and long mental health interventions in children (2,3) and in detecting individual-level symptom improvement at clinical services (4).

**The Moods and Feelings Questionnaire (MFQ)** assesses the presence of affective and cognitive symptoms of depression with items rated on a 3-point Likert about experiences over the course of the last two weeks as being ‘true’, ‘sometimes true’, or ‘not true. The original MFQ has good diagnostic accuracy (AUC=0.82), a high internal consistency (α=0.94) and a high test-retest reliability (ICC=0.78) (5). The shorter 13-item version (sMFQ) showed similarly promising psychometric attributes across cultures and settings(6-9). The MFQ provides a valid and reliable option to monitor changes in depression in short cognitive behavioural interventions (10,11). The original authors recommend that cut points be decided by the users related to their circumstance (12).

**The Strengths and Difficulties Questionnaire (SDQ)** is composed of statements which the respondent identifies as either ‘Not True’, ‘Somewhat True’, or ‘Certainly True’ scored respectively 0, 1 and 2 with regards to their experience in the last 6 months. Half the statements are phrased positively, and reverse scored so that a high score indicates greater difficulty. Items of the first four subscales are then summed up to produce a “total difficulty score” ranging from 0-40. An impact supplement component assesse the extent of distress and impairment caused by any identified problem, which improves the accuracy of detection of disorders in individual children

(13).In addition, an externalising score can be created by summing scores from conduct and hyperactivity scales, while an internalising score is calculated by combining the emotional and peer problem scales. For public health and epidemiological uses, these scale function better as amalgamated scales, whereas the four-factor structure of the scale is of greater value in assessing high-risk samples (14). 

The SDQ has strongly established psychometric properties determined in a nationwide epidemiological sample in the UK (15) as well as across diverse cultures around the globe; Germany (16), Netherlands (17), France (18), Australia (19), South Africa (20), Japan (21) and China (22). Being a broad measure, its utility to evaluate clinical effectiveness in specialist health care appeared less satisfactory compared to other measures of narrowed focus, but allows comparison across a wider range of difficulties than most focused measures (23). The SDQ has been used in several cluster RCTs in Ireland to successfully detect changes following short parent and co-parent interventions (24,25), as well as a cluster RCT teaching classroom management (26). For children aged 4-17 years a newer 4-band categorisation of scores has been validated identifying cut-points for all subscales across all respondents in the UK (27).

**KIDSCREEN** is applicable to healthy and chronically ill children and young people aged 8 to 18 years and is available in long (a 52-item and 27-item) as well as short (10-item) versions (28). Although developed as a self-reported measure it is also available as a parent or proxy measure. The 52-item version measures 10- health related quality of life (HRQoL) dimensions: Physical, Psychological Well- being, Moods and Emotions, Self- Perception, Autonomy, Parent Relations and Home Life, Social Support and Peers, School Environment, Social Acceptance (Bullying), and Financial Resources. The 27-item version amalgamates several of these components, excluding bullying, to create a 5-dimension format from which 10-items are derived to develop the shortest form. The responses are recorded on a Likert scale of 5 categories. Summation of scores of all items produces the dimension score which are transformed in to values between 0 to 100 where higher values signify better HRQoL. T scores are available for stratified by age, gender and socio-economic status (29). The 52-item KIDSCREEN has demonstrated acceptable levels of reliability in all 13 European countries that simultaneously developed the scale; Cronbach’s alpha values ranged from 0.76 -0.89 across all 10 dimensions (30). The same study established satisfactory levels of convergent and discriminant validity. There has been demonstrable evidence of its change to sensitivity in a population based longitudinal Spanish study (31) and the 11-year follow up of the renowned German longitudinal study (BELLA study; 32). Furthermore, the KIDSCREEN-10 has been endorsed by the International Consortium for Health Outcomes Measurement as a standardized global measure of well-being in children and adolescents(33). 

**Supplementary Tables**

Table: S1 Comparison of the mean SDQ scores between MyJournE App data sample and the National sample(Newlove-Delgado et al., 2021)

|  | A: MyJournE App data sample |  |  | B: National sample (from 2021 follow-up survey) |  |  | Difference (A-B) |  |  | t statistics | Statistical significance (p) |
| --- | --- | --- | --- | --- | --- | --- | --- | --- | --- | --- | --- |
|  | Mean | ͣ LCL | ᵇUCL | Mean | LCL | UCL | Mean | LCL | UCL |  |  |
| **All** |  |  |  |  |  |  |  |  |  |  |  |
| Mean total difficulty score | 11.4 | 10.5 | 12.3 | 9.6 | 9.2 | 10.0 | 1.8 | 0.9 | 2.6 | 3.9 | <0.001 |
| Mean emotional problem score | 3.6 | 3.3 | 4.0 | 2.5 | 2.3 | 2.6 | 1.2 | 0.8 | 1.5 | 6.5 | <0.001 |
| Mean conduct problem score | 1.6 | 1.5 | 1.9 | 1.4 | 1.3 | 1.5 | 0.3 | 0.05 | 0.5 | 2.3 | 0.002 |
| Mean hyperactivity problem score | 4.1 | 3.8 | 4.5 | 3.6 | 3.5 | 3.8 | 0.5 | 0.2 | 0.9 | 3.1 | 0.002 |
| Mean peer problem score | 1.9 | 1.6 | 2.1 | 2.1 | 1.9 | 2.2 | -0.3 | -0.4 | 0.03 | -1.8 | 0.09 |
| Mean prosocial behaviour score | 7.6 | 7.4 | 7.9 | 8.0 | 7.9 | 8.1 | -0.4 | -0.6 | -0.1 | -3.1 | 0.002 |
| **Boys** |  |  |  |  |  |  |  |  |  |  |  |
| Mean total difficulty score | 9.5 | 8.3 | 10.6 | 9.6 | 9.1 | 10.2 | -0.07 | -1.2 | 1.1 | -1.2 | 0.908 |
| Mean emotional problem score | 3.6 | 3.1 | 4.1 | 2.0 | 1.8 | 2.2 | 1.6 | 1.1 | 2.2 | 6.1 | <0.001 |
| Mean conduct problem score | 1.7 | 1.3 | 2.0 | 1.5 | 1.3 | 1.6 | 0.20 | -0.12 | 0.52 | 1.2 | 0.223 |
| Mean hyperactivity problem score | 4.0 | 3.5 | 4.5 | 4.0 | 3.8 | 4.2 | 0.04 | -0.47 | 0.55 | 0.14 | 0.886 |
| Mean peer problem score | 1.4 | 1.8 | 2.1 | 2.0 | 2.1 | 2.3 | -0.03 | -0.35 | 0.29 | -0.19 | 0.846 |
| Mean prosocial behaviour score | 7.6 | 7.3 | 7.9 | 7.7 | 7.5 | 7.9 | -0.08 | -0.42 | 0.26 | -0.478 | 0.634 |
| **Girls** |  |  |  |  |  |  |  |  |  |  |  |
| Mean total difficulty score | 12.9 | 11.7 | 14.2 | 9.5 | 9.0 | 10.0 | 3.5 | 2.2 | 4.7 | 5.5 | <0.001 |
| Mean emotional problem score | 3.6 | 3.2 | 4.1 | 3.0 | 2.8 | 3.2 | 0.68 | 0.2 | 1.2 | 2.8 | 0.005 |
| Mean conduct problem score | 1.6 | 1.3 | 1.9 | 1.3 | 1.2 | 1.4 | 0.36 | 0.02 | 0.69 | 2.1 | 0.036 |
| Mean hyperactivity problem score | 4.2 | 3.7 | 4.7 | 3.2 | 3.1 | 3.4 | 1.04 | 0.57 | 1.5 | 4.4 | <0.001 |
| Mean peer problem score | 1.9 | 1.6 | 2.3 | 2.0 | 1.8 | 2.1 | -0.03 | -0.34 | 0.29 | -0.19 | 0.846 |
| Mean prosocial behaviour score | 7.6 | 7.3 | 7.9 | 8.3 | 8.1 | 8.4 | -0.66 | -0.33 | -0.99 | -4.00 | <0.001 |

*ͣ LCL= lower confidence limit*                

*ᵇUCL= upper confidence limit*

Table: S2 Comparison of KIDSCREEN scores between MyJournE App sample and European norms(Ravens-Sieberer et al., 2006)

| Parameter | MyJournE App sample |  | European Norms |  | Mean differences (95% CI) | t statistics | Significance (p) |
| --- | --- | --- | --- | --- | --- | --- | --- |
|  | Mean S score | ͣ SD | Mean S Score | SD |  |  |  |
| School environment | 71.2 | 16.6 | 66.39 | 20.90 | 4.78 (2.6-6.9) | 4.30 | <0.001 |
| Social acceptance | 90.4 | 13.9 | 88.90 | 16.53 | 1.5 (-0.3-3.3) | 1.6 | 0.09 |

*ͣ SD=Standard Deviation*
